# Understanding the Relationship Between Chinese Nurses' Attitudes Toward Incontinence-Related Dermatitis and Achievement Motivation Through a Person-Centered Method: A Theory-Driven Cross-Sectional Study

**DOI:** 10.1155/jonm/3381812

**Published:** 2025-10-29

**Authors:** Siyue Fan, Mufen Ye, Xiaoying Tong, Shuangling Kang, Yanni Lin, Nengtong Zheng, Wenting Chai, Mingqi Zhang, Doudou Yu, Liping Yang, Yutao Wang, Lijuan Chen

**Affiliations:** ^1^Nursing Department, Zhongshan Hospital of Xiamen University, School of Medicine, Xiamen University, Xiamen, China; ^2^School of Nursing, Ningxia Medical University, Yinchuan, Ningxia Hui Autonomous Region, China; ^3^School of Nursing, Fujian University of Traditional Chinese Medicine, Fuzhou, Fujian, China; ^4^Department of General Surgery, Zhongshan Hospital of Xiamen University, School of Medicine, Xiamen University, Xiamen, China; ^5^Intensive Care Unit, Zhongshan Hospital of Xiamen University, School of Medicine, Xiamen University, Xiamen, China; ^6^Department of Gastrointestinal Surgery, Zhongshan Hospital of Xiamen University, School of Medicine, Xiamen University, Xiamen, China

## Abstract

**Background:**

Incontinence-associated dermatitis (IAD) is a common nursing challenge in clinical practice, imposing a significant burden on both patients and healthcare providers. Studies have reported that nurses' preventive attitudes toward IAD significantly influence its prevalence, and there may be a potential association between achievement motivation and these attitudes. Previous research on nurses' preventive attitudes toward IAD has primarily focused on overall levels, overlooking potential heterogeneity within the population.

**Aims:**

This study aimed to investigate the heterogeneity in clinical nurses' preventive attitudes toward IAD using a person-centered approach and to identify the influencing factors for different subgroups. A secondary aim was to utilize Self-Determination Theory (SDT) to elucidate the relationship between the identified attitude profiles and nurses' achievement motivation, thereby providing targeted strategies to enhance their preventive attitudes.

**Methods:**

This study selected 1058 clinical nurses from a tertiary hospital in Fujian, China, as research participants from September to October 2024. The study utilized the following instruments: a general information questionnaire, the Attitude Toward the Prevention of Incontinence-Associated Dermatitis Instrument, and the Achievement Motivation Scale. Latent profile analysis (LPA) was employed to identify the latent profiles of nurses' attitudes toward IAD prevention. At the same time, *t*-tests, chi-square tests, and binary logistic regression analysis were conducted to explore the factors influencing nurses' attitudes across different latent profiles.

**Results:**

Two subgroups of nurses' attitudes toward IAD prevention were identified: the low-level group (63.42%) and the high-level, low-personal-responsibility group (36.57%). A significant correlation was found between nurses' attitudes toward IAD prevention and achievement motivation. Nurses with a more positive preventive attitude scored higher on the motivation for success dimension, while those with a less positive attitude scored higher on the motivation to avoid failure dimension. Factors influencing nurses' attitudes toward IAD prevention included position, department, number of participants in wound/ostomy/incontinence care training, satisfaction with the work atmosphere, and achievement motivation scores.

**Conclusions:**

This study revealed heterogeneity in nurses' attitudes toward IAD prevention. Nurses with positive attitudes tended to adopt a success-driven approach, while those with relatively negative attitudes leaned toward a failure-avoidance strategy, reflecting two fundamentally distinct coping mechanisms.

**Implications for Nursing Management:**

Nursing managers should address these individual differences by targeting achievement motivation as an intervention point. Management strategies should be tailored to the distinct profiles; for instance, interventions for the “low-level group” should prioritize building competence through structured training, while strategies for the “high-level, low-personal-responsibility group” should focus on enhancing autonomy and personal accountability. By adopting such targeted approaches, managers can more effectively enhance nurses' preventive attitudes, thereby improving care quality and reducing IAD incidence.

## 1. Introduction

### 1.1. The Burden of Incontinence-Associated Dermatitis (IAD) and the Current Prevention Gap

IAD is an irritant dermatitis primarily affecting the gluteal, sacral-caudal, and perianal regions, resulting from prolonged exposure to urine and feces [1]. IAD is characterized by diffuse erythema, often accompanied by or without edema of the skin surface [2, 3].

As one of the most common forms of moisture-associated dermatitis, IAD has become a global health concern, necessitating heightened attention from healthcare professionals toward its prevention and management strategies [4]. The incidence of IAD varies significantly across healthcare settings, with reported rates ranging from 5.6% to 50.0% across various institutions and patient populations [5, 6].

In acute care settings, its prevalence is 19% [7], while in long-term care facilities, it reaches 30% [8]. Studies show that in Australian hospitals, the prevalence of urinary and fecal incontinence is 24%, with 42% of those affected ultimately developing IAD [9]. In China, the prevalence of IAD ranges from 14.0% to 32.6%, considerably higher than in other countries [10]. However, these figures may be underestimated due to the absence of internationally standardized tools for IAD assessment and data collection methods [11].

IAD leads to significant discomfort for patients, including pain, pruritus, and sleep disturbances, which adversely affect their health-related quality of life [4]. The condition also carries a psychological burden, causing anxiety and embarrassment due to the social stigma associated with incontinence. Failure to prevent and manage IAD can lead to more severe complications, such as pressure injuries (PIs), which increase treatment costs, prolong hospital stays, and complicate nursing care [12, 13].

In addition to affecting patients, IAD presents significant challenges to healthcare providers. Nurses, in particular, play a critical role in managing IAD, which demands expertise in skin care and prompt intervention. Inadequate management of IAD not only increases the workload of nursing staff but also contributes to job dissatisfaction and burnout. Moreover, insufficient care can result in prolonged recovery times, placing further strain on healthcare facilities. The economic burden on hospitals is considerable, including higher treatment costs, extended patient stays, and the need for specialized care, all of which adversely impact institutional efficiency and patient satisfaction [14].

In light of the prevalence and detrimental impact of IAD, current research primarily focuses on the development of therapeutic products and preventive strategies [14]. However, there remains a lack of effective structured prevention and management protocols. In China, the preventive measures for IAD are suboptimal, and the lack of systematic management and standardized care processes is a pressing concern; regrettably, IAD only receives attention in a limited number of hospitals [15].

### 1.2. Nurses' Working Attitude and Influencing Factors

Evidence-based clinical guidelines are recognized for their potential to reduce IAD incidence and are closely linked with positive healthcare provider perceptions regarding the prevention of common skin injuries [15, 16]. However, the successful implementation of these guidelines often encounters significant barriers. These can include healthcare professionals' lack of knowledge, unfavorable attitudes, and underdeveloped practical skills [17]. Among these, nurses' attitudes are particularly critical determinants in the effective prevention of IAD [18]. The attitudes of healthcare professionals, whether positive or negative, exert a substantial influence on the quality of care delivered to patients [19]. The Theory of Planned Behavior (TPB), proposed by Fishbein and Ajzen [20], offers a framework for understanding attitudes. TPB defines attitudes as acquired predispositions shaped by an individual's knowledge, behavioral intentions, and emotional responses concerning an object or behavior. A central tenet of TPB is that an individual's attitude toward a specific behavior significantly impacts their likelihood of engaging in that behavior. Individuals who perceive a behavior as leading to positive outcomes are more inclined to develop favorable attitudes, thereby increasing their propensity to undertake supportive actions [21]. Conversely, negative attitudes toward an issue tend to reduce the likelihood of participation in related positive behaviors [22].

A significant body of research indicates that a broad spectrum of factors influences nurses' work attitudes, which can be categorized into four primary domains: individual, professional, organizational, and sociocultural. At the individual level, being of an older age [23], female [24], possessing extensive work experience [25], and being motivated by altruistic values are frequently associated with more positive professional attitudes [24]. At the professional level, a strong sense of professional identity, shaped by both nurses' self-perception and public recognition, serves as a significant promoter of positive attitudes, along with job satisfaction [23]. Conversely, dissatisfaction with career advancement opportunities or external rewards [25], excessive workloads, and prolonged career stagnation, such as remaining in the same position for over 4 years without promotion, are common precursors to negative attitudes [25]. At the organizational level, effective managerial support and comprehensive organizational training programs, particularly those addressing professional ethics, are crucial in fostering positive attitudes [23]. The quality of the work environment itself is also a key determinant, where issues like resource shortages, inadequate equipment, and interpersonal conflicts often lead to adverse sentiments [25]. Notably, the adoption of health information technology also shapes attitudes; poor system design, slow performance, or frequent outages can substantially foster negative perceptions [26]. Finally, at the broader sociocultural level, public perceptions of the nursing profession [23], prevailing cultural norms [27], and the extent of family support collectively contribute to the social context shaping these attitudes [28]. In collectivist cultures, such as that of China, values like teamwork and a strong sense of belonging can positively influence nurses' attitudes [29].

Despite this extensive body of research on general nursing attitudes, studies specifically focusing on nurses' attitudes toward IAD prevention remain limited and often lack depth. Existing research in this niche area suggests that factors such as years of experience, level of educational attainment, and receipt of specialized IAD training significantly influence nurses' attitudes regarding IAD care [30, 31]. Furthermore, in the Chinese nursing context, specific cultural factors may play a role. These can include nurses' traditional perceptions of their role in patient care and adherence to established nursing practices, which might inadvertently delay the adoption of newer, effective IAD prevention strategies [22]. This highlights the need for more culturally sensitive research in this area.

### 1.3. Achievement Motivation in Nursing

Achievement motivation is defined as the underlying impetus that drives an individual's pursuit of success and fosters their resilience in the face of adversity [32]. It is generally understood to comprise two primary components: the motivation to achieve success and the motivation to avoid failure [33]. When an individual's behavior is predominantly driven by the desire for success, it typically manifests as proactive engagement and an aspiration for excellence. Conversely, if actions are primarily propelled by the will to avoid failure, outcomes such as hesitation, anxiety, and withdrawal are more common [34]. In a professional context, achievement motivation is a key predictor of employee engagement, individual career development, and even overall organizational performance [35]. Individuals with high achievement motivation typically possess greater self-assurance to overcome obstacles, are dedicated to achieving optimal work performance, and are relentless in the pursuit of their goals [36]. Furthermore, an individual's sense of self-efficacy, combined with their motivation to pursue success, can encourage them to consciously regulate their behaviors and demonstrate positive self-control [37].

The value of achievement motivation is particularly pronounced within the field of nursing. Research confirms that it not only effectively enhances nurses' self-confidence, professional commitment, and drive to succeed [38, 39], but also acts as a crucial catalyst for stimulating their work engagement and increasing job satisfaction. For example, a study involving male nurses in Taiwan revealed that occupational factors such as workload and quality of work life positively predicted their achievement motivation. This study also indicated that achievement motivation can act as a moderating variable, buffering the impact of work-related factors on burnout, particularly within supportive work environments [40]. Therefore, achievement motivation is not only a key psychological factor in shaping nurses' work intentions and behaviors but is also regarded as a promising target for intervention, capable of preventing burnout by elevating nurses' motivational levels [41].

Nurses' achievement motivation is comprehensively influenced by a complex array of factors. These include workplace characteristics (e.g., social support within teams, positive team spirit), work conditions (e.g., hours, compensation, and training opportunities), personal traits (e.g., age, work experience, and educational background), personal priorities (e.g., individual needs and values), and psychological factors (e.g., the perceived meaning of work, an understanding of work outcomes, and a sense of responsibility) [42]. This level of motivation ultimately manifests directly in the quality of nursing services delivered. Studies have demonstrated that nurses with high achievement motivation are more inclined to fully dedicate themselves to patient care, engage in effective teamwork, and exhibit stronger capabilities in behavior, communication, and achieving positive outcomes [43]. Conversely, low levels of motivation often lead to insufficient work engagement and decreased job satisfaction. This can, in turn, cause a decline in the quality of care, reduce patients' likelihood of seeking subsequent care, and may even increase overall healthcare costs [44].

### 1.4. Working Motivation of Nurses Under the Background of Chinese Culture

Nurse motivation is fundamental to ensuring high-quality healthcare services and maintaining healthcare system stability [45]. While certain motivational factors are universal, the cultural context of Asia, particularly China—shaped by regional cultural values, socioeconomic development, and specific healthcare system characteristics—offers a unique perspective on this issue [46]. In the Chinese context, nurse motivation is propelled by a confluence of intrinsic factors, such as altruism, a sense of achievement, and professional growth, alongside extrinsic factors like the work environment and social support [47, 48]. Core work values among Chinese nurses, deeply influenced by prevailing local culture and socioeconomic trends, prominently include altruism, caring, integrity, dignity, responsibility for professional development, autonomy, and justice [49, 50]. Specifically, altruism and caring are widely recognized as essential professional values, with nurses frequently citing the profound sense of accomplishment derived from witnessing patient improvement as a key intrinsic motivator [51, 52]. Despite challenges historically prevalent in the Asian context, such as lower social status and heavy workloads, the motivation drawn from positively impacting patients' lives and the inherent value placed on caring behavior itself remain potent and enduring drivers for many nurses, crucial for workforce stability and retention [53].

Beyond these individual and professional values, the pervasive spirit of collectivism, a characteristic of many Asian cultures, further shapes nurse motivation in China by emphasizing group identity, interdependence, and loyalty, which profoundly impacts teamwork dynamics and organizational commitment [54]. For instance, China's “collectivist spirit” was observed to significantly strengthen nurses' organizational commitment and reinforce their intrinsic work values during national crises like the COVID-19 pandemic [49]. Within the broader Asian nursing environment, collectivism can enhance team cohesion and foster a shared sense of responsibility [55], and research indicates its positive contribution to organizational commitment in China, where harmonious interpersonal relationships are highly valued [56]. However, it is also acknowledged that a strong emphasis on collectivism might, in certain situations, suppress individual opinions, particularly within organizational settings that lack supportive or inclusive leadership styles [57].

### 1.5. Motivation From a Self-Determination Theory (SDT) Perspective

SDT, widely applied across diverse fields including education and healthcare [58, 59], was developed by Deci and Ryan [60] to elucidate the roles of autonomous and controlled dimensions of motivation. SDT posits that the intensity and persistence of individual behavior are significantly dependent on the satisfaction of certain basic psychological needs (BPNs). It fundamentally categorizes motivation into intrinsic motivation, arising from internal interests or inherent satisfaction, and extrinsic motivation, driven by external pressures or the pursuit of rewards [61].

SDT conceptualizes extrinsic motivation along a continuum of four forms distinguished by their degree of internalization: external regulation, introjected motivation, identified motivation, and integrated motivation [62]. External regulation, the least internalized form, involves behavior controlled by explicit external contingencies such as rewards or punishments [63]. Introjected motivation stems from internalized pressures, where behavior is driven by a desire to avoid guilt or anxiety, or by a sense of obligation, often characterized by a feeling of “should” [64, 65]. Identified motivation occurs when individuals recognize and value behaviors as personally significant and aligned with their own goals, driven more by a sense of “want” than by external dictates [66]. Integrated motivation, representing the most internalized form of extrinsic motivation, arises when the value of a behavior, even if not inherently enjoyable, is fully assimilated into an individual's sense of self and aligns with their other core values and needs [67].

These distinct forms of motivation are further categorized by SDT along a spectrum from autonomous to controlled autonomous motivation, which encompasses intrinsic motivation alongside the more internalized extrinsic forms of identified and integrated motivation, reflects self-directed actions that are consistent with personal values and foster a sense of agency [68]. Conversely, controlled motivation, comprising external regulation and introjected motivation, is typically driven by external pressures or internalized compulsions, often engendering feelings of obligation or passivity and diminishing an individual's sense of autonomy [69]. Research drawing on SDT consistently indicates that autonomous motivation generally fosters more positive attitudes and sustained, high-quality behaviors [70]. Particularly in workplace settings, autonomous motivation, especially when supported by autonomy-enhancing environments, is strongly associated with greater employee well-being, engagement, and performance compared to controlled motivation [71–74]. In contrast, controlled motivation tends to exert a weaker influence on long-term engagement and, in some instances, may even lead to negative outcomes or diminished well-being [70].

At the core of SDT are three fundamental psychological needs essential for optimal functioning and psychological growth: autonomy, competence, and relatedness [68, 75]. The need for autonomy pertains to an individual's desire to act volitionally in accordance with their authentic self, free from external coercion; its satisfaction is critical for fostering intrinsic motivation and self-regulation [68, 75]. The need for competence involves feeling capable and effective in one's actions, serving as a key source of intrinsic satisfaction and a driver of engagement, particularly when accompanied by a sense of autonomy [68]. The need for relatedness concerns the desire for meaningful connections and a sense of belonging [61], which significantly enhances intrinsic motivation in supportive contexts [76] and is vital for the internalization of extrinsically motivated behaviors [77], especially when these are valued by significant, respected others [78]. Consistent with this, intrinsic motivation, fueled by need satisfaction, is strongly associated with the pursuit of success, whereas certain forms of extrinsic motivation may correlate more with a fear of failure [79–82].

While SDT has been widely applied to examine various aspects of nursing practice, including learning behaviors and professional commitment [59, 83–86], its specific application to understanding nurses' attitudes toward IAD prevention remains underexplored. Building on SDT, we hypothesize that nurses who are autonomously motivated by intrinsic values, such as the desire to improve patient outcomes, will exhibit proactive IAD prevention attitudes. In contrast, those with predominantly controlled motivation, propelled by external pressures like avoiding penalties, may display reluctance. This aligns with SDT's emphasis on autonomy, defined here as perceiving decision-making agency regarding IAD prevention, and competence, understood as believing in one's knowledge and skills for its management, as pivotal drivers of sustained positive behaviors. Furthermore, a supportive team environment that fosters relatedness is hypothesized to contribute positively. Consequently, the fulfillment of these three BPNs is considered integral to fostering the proactive attitudes indicative of autonomous motivation in the context of IAD prevention.

### 1.6. Person-Oriented Versus Variable-Oriented Approaches

Person-centered research is characterized as “a holistic, interactive perspective, in which the individual is viewed as an organized whole, functioning and developing as a unit [87].” This approach contrasts sharply with the variable-centered perspective, which aims to assess relationships between variables across individuals. Consequently, a key distinction lies in their starting points and focus: The person-centered perspective first seeks to identify distinct subgroups of individuals who share a common profile of attributes and then describes how these homogeneous groups function. In contrast, the variable-centered perspective typically begins by identifying variables of interest and subsequently describes how these variables operate and interrelate across the entire sample [71, 87].

Previous research concerning nurses' attitudes toward preventing IAD has predominantly employed a variable-centered approach. This approach primarily focused on assessing the status quo of these attitudes, thereby overlooking the variability attributable to individual differences. Previous studies have reported varying findings regarding the average level of nurses' attitudes toward IAD prevention. Mengying's study found clinical nurses' preventive attitudes toward IAD were at a medium level [88]. Wang et al. surveyed clinical nurses in Henan Province, China, using the IAD Knowledge, Attitude, and Practice questionnaire, and reported relatively high levels of attitudes [89]. In contrast, Li et al., using a self-developed questionnaire, found ICU nurses in Hainan Province, China, exhibited less favorable attitudes toward IAD [90]. A significant limitation of these prior studies was their focus on the average level of nurses' attitudes toward IAD prevention. This focus implicitly assumed homogeneity within the nursing population and consequently failed to account for potentially significant differences among nurses with varying characteristics. Thus, to some extent, the findings from these studies may not fully represent the diversity within the nursing population.

Addressing these limitations and exploring potential heterogeneity, latent profile analysis (LPA)—a sophisticated person-centered statistical technique—offers several distinct advantages for this research context. Firstly, LPA enables the identification of underlying heterogeneity within the study population by classifying individuals into distinct, unobserved subgroups or “latent profiles” based on their patterns of responses to a set of indicators [91, 92]. Nurses within each identified preventive attitude subgroup are expected to be relatively homogeneous, while those across different subgroups will exhibit heterogeneity. This approach can provide a much richer understanding of the distinct characteristics of nurse populations with varying preventive attitudes. Secondly, LPA can help reduce the complexity often associated with higher-order interactions of variables encountered in variable-centered methods. Traditional methods might require larger sample sizes to detect such interaction effects and often leave uncertainty regarding how multiple variables combine within individuals to form distinct patterns [93, 94]. Thirdly, LPA has been successfully applied in numerous psychological trait studies, demonstrating its utility in uncovering meaningful person-centered patterns. The identification of such distinct profiles can be invaluable for developing tailored interventions designed to meet the unique needs of specific subgroups [95, 96].

### 1.7. Research Objectives

Therefore, this study aims to explore the heterogeneity in nurses' attitudes toward IAD prevention using a person-centered analytical approach. Specifically, LPA was applied to survey data from Chinese nurses to identify distinct latent profiles based on their responses to the IAD prevention attitude questionnaire. Subsequently, guided by SDT, we examined whether nurses' individual characteristics and achievement motivation differed significantly across these identified IAD prevention attitude profiles. The application of SDT in this study serves three primary purposes: (1) to formulate core hypotheses regarding the relationship between nurses' achievement motivation orientations and their attitudes toward IAD prevention; (2) to provide a robust theoretical framework for interpreting the statistical findings, particularly in the discussion section, by elucidating how the satisfaction or frustration of BPNs mediates the influence of demographic and work-related factors on nurses' attitude profiles; and (3) to develop precisely targeted, theory-driven nursing management interventions tailored to the specific psychological needs of distinct nurse subgroups. In summary, this study employs LPA to adopt a person-centered perspective, utilizing SDT to gain deeper insights into the heterogeneity of nurses' IAD prevention attitudes and their underlying motivational mechanisms. The findings are expected to provide novel perspectives on nurses' IAD prevention attitudes and highlight the value of personalized interventions based on person-centered assessments. Understanding the heterogeneity in nurses' IAD prevention attitudes may contribute to reducing the clinical incidence of IAD, while the results could inform the development of tailored intervention strategies.

### 1.8. Hypotheses

**Figure 1:**
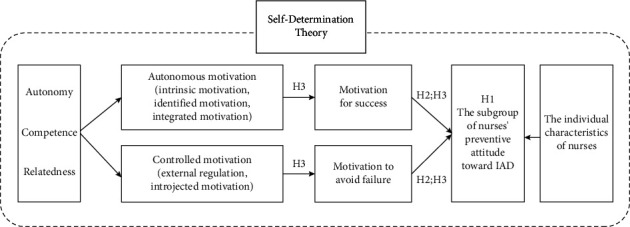
Conceptual framework diagram.

Drawing on the postulates of SDT, we propose that nurses with differing motivational orientations exhibit distinct attitudes toward IAD prevention. The conceptual framework diagram is shown in Figure 1. Specifically, we hypothesize that

  H1: There are subgroup differences in nurses' attitudes toward IAD prevention.
  H2: Nurses' attitudes toward IAD prevention are associated with their achievement motivation.
  H3: Nurses with higher autonomous motivation (success-driven) will report more positive IAD prevention attitudes, whereas those with controlled motivation (failure-avoidant) will exhibit less proactive attitudes.

## 2. Materials and Methods

### 2.1. Design

A cross-sectional study was carried out in 2024 between September and October. The Strengthening the Reporting of Observational Research in Epidemiology (STROBE) statement was adhered to in this study (see Appendix S1) [97].

### 2.2. Participants and Setting

Participants were nurses conveniently sampled from a large, tertiary comprehensive hospital located in Xiamen City, Fujian Province, China. This specific hospital was selected for several reasons. Firstly, it is considered representative of major public tertiary care facilities in the region, characterized by a diverse range of clinical departments and a substantial nursing staff, which facilitated the recruitment of a large and varied sample. Secondly, the hospital holds a significant role in specialized nursing care; it serves as a national-level training base for specialist nurses in wounds, ostomies, and IAD and is the main unit for the Xiamen City Wound, Ostomy, and Incontinence Nursing Committee in Fujian Province, signifying a high level of expertise in these specialized areas within the region.

Despite this recognized specialist training capacity, the current study aimed to assess the IAD prevention attitudes of the broader population of general clinical nurses. During the study period (September–October 2024), the hospital adhered to standard Chinese IAD prevention guidelines applicable to tertiary care. Beyond routine nursing education and standard protocols, no additional hospital-wide specialized or advanced IAD management programs, distinct from those in other similar tertiary facilities following national standards, were universally implemented for all general nursing staff. This context was deemed suitable for examining the spectrum of general nursing attitudes toward IAD prevention.

To assess the representativeness of the sample, key demographic characteristics were compared with national data for registered nurses from the “2023 China Health Statistics Yearbook” (China National Health Statistics Information Center). This comparison revealed that the study sample exhibited similarities with the national nursing population in terms of gender distribution, age structure, average years of service, and the distribution of professional titles. However, a notable difference emerged in educational attainment: Nurses in this study had a markedly higher level of education, with 1.8% holding master's degrees or above (compared to 0.4% nationally) and 80.2% holding bachelor's degrees (compared to 37.0% nationally). Correspondingly, the proportion of nurses with Junior College diplomas or below was lower in this study's sample (18.0%) than the national figure (62.4%).

Inclusion criteria were as follows: (a) completion of nurse registration and the National Nurse Licensure Examination (NNLE); (b) independent performance of nursing duties; and (c) willingness to participate in the study. Nursing students and nurses absent due to illness or leave during the study period were excluded.

### 2.3. Sample Size

The sample size was calculated as 10 times the number of scale items, following Kendall's sample size estimation standard [98]. This study included 44 items: the Chinese version of the Attitude Toward the Incontinence-Associated Dermatitis Scale (14 items) and the Achievement Motivation Scale (AMS) (30 items). Therefore, the minimum required sample size was *N* = 10 ∗ (30 + 14) = 440. Considering a 10%–20% nonresponse rate, the final required sample size was *N* = 440 ÷ (1%–20%) = 550.

This calculation established the minimum threshold for the study. However, to enhance statistical power and the stability of the LPA, which typically benefits from larger sample sizes [99, 100], the study aimed to recruit as many eligible participants as feasible from the selected large tertiary comprehensive hospital. Therefore, during the data collection period (September–October 2024), all nurses meeting the inclusion criteria within the accessible departments of the hospital were invited to participate. This recruitment strategy resulted in the collection of 1058 complete and usable questionnaires, substantially exceeding the initially calculated minimum requirement. This larger achieved sample size provides a robust dataset for the planned statistical analyses, particularly for ensuring the reliability and interpretability of the LPA results.

### 2.4. Instrument

#### 2.4.1. Demographic Questionnaire

A demographic and work-related characteristics questionnaire was compiled for this study. The selection of items was based on a comprehensive review of relevant literature to identify key demographic, professional, and work environment factors potentially associated with nurses' attitudes and motivation, as well as the specific objectives of this research. The questionnaire gathered information on: gender, age, professional title, position, specialized nurse status, highest educational attainment, department, years of nursing experience, membership in a wound/ostomy/incontinence care team, history and type of wound/ostomy/incontinence care training received, number of study visits per year, and single-item ratings of satisfaction with the working environment, work atmosphere, and overall job satisfaction.

#### 2.4.2. Attitude Towards the Prevention Incontinence-Associated Dermatitis Instrument (APrIAD)

The APrIAD is employed to assess nurses' attitudes toward the prevention of IAD, a tool developed by Van Damme et al. [18] and adapted into Chinese by Jin [101]. This instrument comprises 14 items clustered into four dimensions reflecting key theoretical themes:

a. Perceptions regarding the impact of IAD on patients (5 items): Assesses beliefs about the severity and consequences of IAD for patient well-being.
b. Beliefs about the team's responsibility in preventing IAD (3 items): This dimension gauges perceptions of collective accountability within the nursing team and the influence of team norms and colleague behaviors (e.g., learning from experienced role models) on IAD prevention practices. It reflects the understanding that IAD prevention is often a shared effort influenced by the team environment.
c. Convictions about personal responsibility in preventing IAD (3 items): This dimension focuses on the individual nurse's belief in their own capacity to make a difference in IAD prevention and their sense of personal accountability. It reflects the conviction that their own nursing care quality directly impacts IAD development and their individual duty to apply preventive measures.
d. Opinions on the efficacy of IAD prevention products and procedures (3 items): Assesses beliefs about the effectiveness of available interventions and products for preventing IAD.

The key distinction between the “team responsibility” and “personal responsibility” dimensions lies in the focus: “Team responsibility” emphasizes shared accountability, collective norms, and systemic influences within the team, whereas “personal responsibility” centers on the individual nurse's sense of duty, perceived efficacy, and direct contribution to prevention.

A 4-point Likert scale is utilized, with scores ranging from 0 to 3, representing “strong disagreement” to “strong agreement,” and a total score of 0–42, where higher scores indicate a more positive attitude toward IAD prevention. The scale exhibiting good data fit (*χ*^2^ = 2810.769, *χ*^2^/df = 2.197, CFI = 0.941, GFI = 0.936, CFI = 0.941, NFI = 0.921, RMSEA = 0.037) was subjected to a confirmatory factor analysis in previous studies. Chinese populations have validated the Scale. In Yu's [102] and Mengying's et al. [88] research, Cronbach's *α* was 0.774 and 0.809, respectively; in this study, it was 0.766 for the nursing environment.

#### 2.4.3. AMS

The AMS scale is utilized to measure nurses' achievement motivation. This scale was developed by Nygård and Gjesme [103] in 1973 and revised into Chinese by Chinese scholars Ye and Hagtvet [104]. It comprises two dimensions: the motivation for success (MS) (with 15 items) and the motivation to avoid failure (MF) (with 15 items), totaling 30 items. The Likert 4-point scoring system is employed, where scores from 1 to 4 correspond to “*Strongly Disagree*” to “*Strongly Agree*.” The total score for each dimension ranges from 15 to 60 points. Achievement motivation score is calculated as the difference between the MS score and the MF score, with higher scores indicating stronger achievement motivation.

Previous studies utilizing the Chinese version of the AMS within various Chinese nursing populations have provided evidence supporting its psychometric properties. For instance, research involving resident nurses reported a split-half reliability coefficient of 0.77 [105]. Studies with emergency department nurses found good internal consistency, with a Cronbach's alpha coefficient of 0.896 reported [106]. Furthermore, research among operating room nurses indicated acceptable to good internal consistency, with Cronbach's alpha coefficients ranging from 0.85 to 0.87, and moderate retest reliability (coefficient = 0.62) over a specified period [107]. These findings suggest the scale's suitability for assessing achievement motivation in diverse Chinese nursing contexts. In the present study, the overall Cronbach's alpha coefficient for the AMS was 0.903.

### 2.5. Data Collection

The information was gathered through a self-reported, anonymous questionnaire. Using an online self-reported questionnaire, a presurvey was conducted with 10 registered nurses in this hospital before the informational survey to determine whether the questions were understandable and whether there were any technical issues (these data were not incorporated in this study). The researchers were responsible for liaising with the leaders of the wound care teams in each department, explaining the purpose of the survey and the method for completing the questionnaire. With the consent and assistance of the departmental administrators, a link to an anonymous electronic questionnaire was sent via WeChat to the leaders of the respective wound care teams, who then distributed it to the clinical nurses within their departments. At the start of the questionnaires, permission and explicit instructions were given. The informed consent process was integrated into the online questionnaire. Prior to accessing the survey items, an information sheet was presented on the first page. This sheet provided comprehensive details regarding the study's purpose, the nature and expected duration of participation, the voluntary basis of involvement, and an assurance that participation or nonparticipation would not affect their employment. It also outlined the procedures for ensuring the anonymity of responses and the confidentiality of the collected data, how the data would be securely stored and used for research purposes only, and provided contact information for the research team should participants have any questions or concerns. Participants indicated their informed consent by acknowledging this information and actively agreeing to proceed with filling out and subsequently submitting the questionnaire. To ensure data completeness, the online questionnaire system was configured to require responses to all items; participants could not submit the questionnaire if any items were left unanswered, as prompts would direct them to the incomplete sections. This design ensured that all submitted questionnaires were fully completed in terms of item responses. This rigorous process ensured high data quality. Two investigators with consistent training kept an eye on the recovery data at all times. The data were exported after a week in which no new data were produced. Following data collection, an initial data screening was conducted by two researchers. During this process, any questionnaires that did not meet predefined quality completion standards (e.g., exhibiting invariant responses across all items, patterned responses inconsistent with genuine engagement, or those deemed highly unrealistic based on completion time or response contradictions) were identified and excluded from the final dataset. This quality screening led to the removal of 4 questionnaires. Ultimately, 1058 complete and valid questionnaires were deemed suitable and utilized for the analyses. This rigorous process ensured high data quality. In this study, no materiality-based reward was provided.

### 2.6. Data Analysis

For data analysis, this study employed Mplus 8.3 and SPSS 26.0. Kolmogorov–Smirnov was used to test the normality of the measurement data, and the enumeration data were expressed as *n* (%). Standard deviation (SD) and mean were used to represent normal data, whereas median and interquartile range were used to represent skewed data. The scores of each dimension of nurses' prevention attitude toward IAD were the main variables in our LPA, which we performed after first applying the normality and common method bias (CMB) tests to the data. LPA is a statistical technique designed to evaluate individual characteristics based on multiple continuous variables. It uses probabilistic methods to determine the subgroup to which an individual most likely belongs, identifying patterns to categorize individuals into distinct groups. To determine the optimal number of latent profiles, models with one to four profiles were estimated and compared. The selection of the optimal model was guided by a combination of statistical fit indices, theoretical interpretability, class size, and classification accuracy. Key fit indices examined included the Akaike Information Criterion (AIC), Bayesian Information Criterion (BIC), and sample-size adjusted BIC (aBIC), where lower values 1 generally indicate better relative model fit. Additionally, the Vuong–Lo–Mendell–Rubin likelihood ratio test (VLMR-LRT) and the bootstrapped likelihood ratio test (BLRT) were used to assess whether a k-class model provided a statistically significant improvement over a k-1 class model. Entropy values were examined for classification precision, with values closer to 1 indicating clearer class distinction, and class solutions were also evaluated based on the practical requirement that each class should comprise at least 5.0% of the total sample for meaningful interpretation, as shown in Table 1 [100, 108–110]. Once the ideal number of latent profiles was determined, nurses were assigned to latent profile groups based on their most probable latent class membership. To identify statistically significant differences among the preventive attitude subgroups, univariate analyses, including the *t*-test and chi-square test, were first conducted. Variables that were significant in the univariate analyses (*p* < 0.05, two-sided) were included in the binary logistic regression model. The cutoff for inclusion in the final model was set at *p* < 0.05.

### 2.7. Common Method Deviation Test

There can be some common method variance (CMV) because this study used self-report data [111]. The reverse item approach, an anonymous questionnaire, and randomization of item assignment across different constructs were among the strategies we employed in this study to prevent CMV.

In this study, the effects of an unmeasured latent methods factor (ULMC) were controlled to investigate the existence of CMB. A bifactor model that included a common method factor was tested based on the original model. Confirmatory factor analysis results showed that adding the common method factor to the original model resulted in negligible modifications (< 0.05) in several fit indices (ΔRMSEA = 0.140, ΔSRMR = 0.113, ΔCFI = 0.642, ΔTLI = 0.595). We discovered that there is no significant CMB since the model did not considerably refine the fitting effect by controlling the common method components.

### 2.8. Ethical Considerations

Ethical approval for this work was provided by The Research Ethics Committee of Xiamen University's Zhongshan Hospital (Approval No: xmzsyyky(2024-055)).

## 3. Result

### 3.1. Participant Characteristics

The study sample consisted of 1058 nurses, with a predominantly female population (96.2%, *n* = 1018) and a smaller proportion of males (3.8%, *n* = 40). The majority of nurses were between 26 and 35 years old (49.4%, *n* = 523). In terms of job roles, 52.8% (*n* = 559) held supervisory positions, while 91.7% were clinical nurses. Wound ostomy continence specialist nurses accounted for only 0.8% (*n* = 8) of the sample, with 13.1% (*n* = 139) representing other specialized nurses. The highest qualification for most nurses was a bachelor's degree (80.2%, *n* = 849). Departmentally, 46.5% (*n* = 492) of nurses worked in medical departments, while 40.2% (*n* = 425) were from surgical departments. The largest proportion of nurses had 11–20 years of work experience (38.5%, *n* = 407), followed by those with 6–10 years of experience (25.9%, *n* = 274). Further details on participant characteristics are provided in Table 2.

### 3.2. Results of LPA

Four latent profile models in all were explored in this work, and Table 3 shows the fitting indexes of the various profiles. The values of AIC, BIC, and aBIC rapidly decreased as the number of latent profiles increased. When the profile was divided into three categories, the VLMR-LRT was not statistically significant, suggesting that the two-profile and four-profile models performed better than the three-profile model. Since each category should make up at least 5.0% of the entire sample size, the four-profile model's profile had a very small proportion, which made it impractical to use. Contrary to this, Model 2's entropy value is the highest. In Model 2, the two categories had average attribution probabilities of 98.7% and 97.4%, respectively (refer to Table 4). The category model's VLMR-LRT and BLRT were statistically significant, suggesting that Model 2 was the best fit. Given the extensive analysis described above, the best classification result for clinical nurses' attitudes toward IAD prevention was obtained by using a two-class solution.

### 3.3. Naming Categories of Latent Profiles of Nurses' Attitudes Toward IAD Prevention

**Figure 2:**
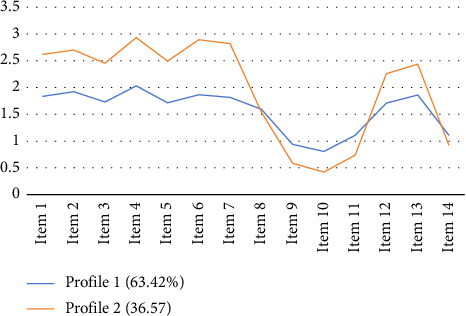
Distribution of two latent profiles of IAD prevention attitudes.

A chart was created based on the scores of the two nurse categories' attitudes toward IAD prevention on the 14 items, and Model 2 was chosen as the best model to identify the categories based on the scores of the two nurse categories on the 14 items (see Figure 2). Profile 1 accounted for 63.42% of the total, and because of its low item scores, it became known as the low-level group. Profile 2, with a share of 36.57%, had the highest scores for most items in this category. Among them, Item 8 (“I invest more time in preventing IAD compared to my colleagues”), Item 9 (“I will inform the responsible medical doctor if a patient suffers from IAD”), Item 10 (“If many patients suffer from IAD, I will discuss this with the senior nurse”), Item 11 (“I will discuss each IAD case with my colleagues”), and Item 14 (“IAD incidence is an indicator of the quality of care”) scored the lowest. Among these items, Items 9, 10, and 11 belong to the dimension of personal responsibility, reflecting the fact that such nurses believe that it is difficult for personal behavior to make a difference in the prevention of IAD. Therefore, it was named as high-level low-personal-responsibility group.

### 3.4. Descriptive Statistics and Correlations

There exists a statistically significant disparity in the average scores of the four dimensions of the IAD prevention attitude across two latent profiles (*p* < 0.001), as evident from Table 5. The average scores of individual items in the IAD prevention attitude are presented in Table 6. There was also a significant difference between the latent profile of nurses' attitudes toward IAD prevention and their mean scores on achievement motivation (*p* < 0.001), with Profile 1 demonstrating a higher average score in the MF dimension and Profile 2 exhibiting a higher average score in the MS dimension (see Table 7). Furthermore, the Pearson correlation analysis revealed a strongly positive correlation between the IAD prevention attitude and achievement motivation (*r* = 96%, *p* < 0.05) (see Table 8).

### 3.5. Factors Influencing Nurses' Attitudes Toward IAD Prevention

The results of the univariate analysis revealed significant differences in several variables across the two latent profiles: professional title, position, department, years of nursing work, participation in wound/ostomy/incontinence care training, training frequency, training method, work environment satisfaction, work atmosphere satisfaction, job satisfaction, and achievement motivation (see Table 9).

### 3.6. Binary Logistic Regression of Factors Influencing Nurses' Attitudes Toward IAD Prevention

The two latent profiles identified through LPA were employed as dependent variables, while the variables demonstrating statistically significant differences in univariate analysis were utilized as independent variables in a binary logistic regression analysis. The coding of each variable is presented in Table 10. The results revealed that position, department, number of training attended, satisfaction with work atmosphere, and achievement motivation were influential factors affecting nurses' preventive attitudes toward IAD (See Table 11).

## 4. Discussions

The nurses' attitudes toward IAD prevention exhibit considerable heterogeneity, a crucial finding that emerges from the adoption of novel methodologies. Previous studies have characterized nurses' preventive attitudes at an aggregate level; while the findings and implications of these studies are by no means negligible, their limitations overlook the potential for stratification. This study employed a person-centered approach to identify heterogeneous attitudes toward IAD prevention among Chinese nurses. It further examined the association between demographic factors and identified attitude profiles and explored the relationship between these latent categories of IAD prevention attitudes and achievement motivation. To our knowledge, this is the first study to investigate nurses' attitudes toward IAD prevention using this methodological approach.

### 4.1. Potential Profile Characteristics of Chinese Nurses' Attitudes Toward IAD Prevention

Clinical nurses' attitudes toward IAD prevention were found to fall into two profiles using LPA: the low-level group and the high-level low-personal-responsibility group, which accounted for 63.42% and 36.57% of the total, respectively. Nurses in the high-level group exhibited a higher prevalence of senior titles, 11–20 years of experience, and six or more training sessions compared to their low-level counterparts. Conversely, nurses in the low-level group were more likely to hold junior titles, have 6–10 years of experience, and undergo 1 to 2 training sessions. Studies indicate that the length of working years is closely linked to clinical experience and professional title promotion [112]. Nurses with extensive clinical practice are proficient in identifying and mitigating potential safety risks associated with IAD, adopting a proactive approach to its prevention and early resolution. Promotion in nurse professional titles requires sufficient working years and the successful completion of relevant examination of professional-related knowledge and skills [113]. Nurses holding higher professional titles, having accrued significant professional knowledge through extended periods of service, possess a clear understanding of the risks IAD poses to patients and nursing care. Conversely, nurses with junior professional titles have less experience, which must be incrementally acquired [114]. They often lack adequate knowledge about IAD, fail to appreciate its dangers fully, and do not possess the necessary skills to assess and manage IAD effectively, resulting in a less proactive attitude toward its prevention.

In this study, Chinese clinical nurses' average score for their attitude toward IAD prevention was 24.17 ± 4.83, with a score rate of 57%, which was at a moderate level. Notably, 671 nurses exhibited a positive attitude, achieving a higher average score of 27.81 ± 3.61. Conversely, 387 nurses demonstrated relatively less positive attitudes, with a lower average score of 22.02 ± 4.12. Compared to another study focusing on Chinese ICU nurses, the scores in our research were lower, potentially attributable to the higher prevalence of critically ill patients in ICUs, the substantial workload of nurses, and their enhanced ability to identify special skin issues [88]. Further insights emerge from an examination of responses to specific APrIAD items. Notably, Item 8 (“I invest more time in preventing IAD compared to my colleagues”) and Item 14 (“IAD incidence is an indicator of the quality of care”) received relatively lower scores across the sample. This may suggest that while IAD is recognized, its status as a key nursing quality indicator is not universally internalized, or that nurses perceive constraints (e.g., time, staffing) on their ability to dedicate exceptional individual effort to its prevention compared to peers, possibly reflecting the collectivistic or systemic pressures [115, 116]. The concept of IAD itself, though globally established as preventable and treatable [116], might still be variably understood or prioritized among clinical nurses, leading to differences in cognitive and evaluative approaches [117–119].

An intriguing finding in our study is that nurses in the “high-level” profile exhibited lower scores on the personal responsibility dimension compared to those in the “low-level” profile. This phenomenon may be attributable to a confluence of factors, including pervasive cultural influences, entrenched hierarchical organizational structures, a tendency toward the externalization of responsibility, potentially insufficient role clarity or a circumscribed understanding of their professional role, an over-reliance on institutionalized procedures, and aspects of the broader work environment [114, 120].

Chinese culture is profoundly shaped by collectivistic values, which emphasize group harmony, interdependence, and the fulfillment of responsibilities and obligations toward family and society at large [48]. Research indicates that cultural and socioeconomic trends significantly influence nurses' comprehension and interpretation of their professional values, including their sense of responsibility [121]. Within China, societal work ethics, rooted in Confucian philosophy, prioritize collective well-being over individual needs, a perspective that contrasts with the emphasis on individual accountability often found in Western individualistic cultures [122]. In this collectivistic milieu, the “relatedness” dimension of SDT assumes particular prominence [123]. Nurses' sense of responsibility may, therefore, manifest more as an alignment with and contribution to the collective goals of the team, department, or even the entire hospital, rather than solely as the independent assumption of individual duties [124]. Existing studies suggest that even when initial motivation is not highly autonomous, a culturally endowed sense of responsibility can still drive nurses' behavior [125]. Within the Chinese cultural framework, fulfilling the expectations of the collective or others—an expression of the need for relatedness—can itself be a source of positive psychological experience, even if it entails some compromise of individual autonomy. Consequently, the “low personal responsibility” observed in some nurses within the “high-level” profile may not signify an absence of responsibility per se. Instead, it could represent an adaptive expression of responsibility that prioritizes the maintenance of group harmony and the satisfaction of relational needs, molded by collectivistic cultural influences. Their sense of accountability might be more oriented toward collective objectives and overall team performance, rather than an emphasis on individual initiative and independent burden-bearing, particularly if individualized assumption of responsibility risks disrupting existing team equilibrium or contravening collective expectations. This phenomenon could be interpreted not as a deficiency in responsibility, but as an adaptive articulation of it within a specific cultural context.

Furthermore, hierarchical systems are a significant contextual factor, as Chinese hospitals commonly feature pronounced hierarchies that profoundly impact nurses' autonomy and their sense of personal responsibility [125]. Studies have indicated that Chinese nurses may experience “decision-making constraints” and might even require permission before undertaking certain autonomous nursing practices [126]. Concurrently, nurses often report feeling “undervalued” by physicians and managers, reflecting power imbalances within the healthcare team [125]. In accordance with SDT's concept of “autonomy,” if nurses' clinical decision-making authority is severely restricted within a rigidly hierarchical hospital environment, and their scope for professional judgment and autonomous action is compressed, then their sense of “ownership” over work tasks and patient outcomes and consequently their personal responsibility may be attenuated [127]. When decisions are predominantly made by superiors, nurses may perceive themselves primarily as executors of directives rather than as professionals bearing primary responsibility for outcomes, thereby fostering an externalization of responsibility. Within such hierarchical structures, the locus of accountability often shifts upwards. Nurses might believe their foremost duty is the precise execution of superiors' orders, rather than proactively identifying problems, independently managing risks, or autonomously resolving issues. This behavioral pattern can be viewed as an adaptive response under conditions of “external regulation.” While this may, to some extent, satisfy nurses' need for “competence” in specific tasks, it significantly impedes the exercise of their autonomy concerning broader patient care outcomes, potentially contributing to a less developed sense of their comprehensive professional role [128]. This tendency to attribute primary responsibility to decision-makers is a rational adaptation by individuals to mitigate risks and navigate a controlled environment [129, 130].

Considering the interplay of Chinese collectivistic culture and hospital hierarchical structures offers a more comprehensive explanation for the observation of “low personal responsibility” among some “high-level” nurses. The confluence of these two factors may cultivate a work environment where even experienced or senior nurses might exhibit reluctance to assume personal risks, a preference for collective (even if less efficient) decision-making, or a mode of practice strictly adhering to protocols rather than flexibly applying professional judgment, especially when individual actions could challenge authority or disrupt team cohesion.

In many Western healthcare systems, such as those in the United States or parts of Europe, a deep-rooted cultural and professional emphasis is placed on individual responsibility and accountability [131, 132]. Healthcare professionals are typically expected to be personally answerable for their actions, clinical decisions, and ethical conduct, a principle considered foundational to ensuring patient safety, maintaining quality of care, fostering patient trust, ensuring legal compliance, and enhancing staff morale [133]. Professional ethical codes further underscore this, emphasizing that even within collaborative team environments, individuals bear responsibility for fulfilling their duties and adhering to standards of conduct; a culture of direct ownership over tasks and outcomes is actively encouraged. Furthermore, even as collective leadership and team-based care models emerge, a tension can exist with traditional legal frameworks that often assign ultimate legal and professional responsibility to specific individuals [134]. Despite the high value placed on teamwork, the final locus of accountability in these systems frequently rests with the individual, reflecting broader cultural values that emphasize personal agency and direct responsibility [135].

Consequently, were a “Higher General Attitude with Lower Personal Responsibility” profile to emerge prominently in such Western contexts, its interpretation might differ significantly. While systemic factors would invariably be considered, such a profile could more readily be hypothesized as indicative of issues like professional burnout leading to disengagement, a pervasive fear of litigation that paradoxically suppresses overt acceptance of responsibility despite strong underlying norms, or overly rigid organizational processes that stifle individual initiative [135–137]. The prevailing cultural expectation in these settings would generally anticipate a more direct and positive correlation between comprehensive professional engagement and individualized responsibility. This contrasts with the Chinese context, where, as previously discussed, the interplay of collectivistic values and hierarchical structures may uniquely shape the expression of responsibility. This suggests that while the fundamental psychological needs posited by SDT are universal, the sociocultural and organizational environment critically shapes how responsibility is perceived, enacted, and its relationship with general professional attitudes. Future cross-cultural comparative studies employing similar methodologies could further elucidate these nuanced differences in the expression and determinants of nurses' professional accountability and attitudes globally.

### 4.2. Correlations Between Nurses' Attitudes Toward IAD Prevention and Achievement Motivation

In this study, the average score for nurses' achievement motivation was 0.89, lower than that of ICU nurses in China but higher than those in emergency departments and rheumatology and immunology units [88, 106, 138, 139]. A key finding was the difference in achievement motivation components between the identified latent profiles. Nurses in the “Lower Overall Attitude” profile (Profile 1) exhibited a higher average score in the motivation to avoid failure dimension (39.42), exceeding that of the “Higher Overall Attitude” profile (Profile 2). Conversely, in the MS dimension, Profile 2 scored higher (average of 41.88) than Profile 1.

These findings align with the theoretical understanding of achievement motivation. A predominant MS suggests a stronger desire for success, a willingness to engage with novel challenges, and less concern about failure, fostering positive emotions and proactive engagement [40]. Such nurses are likely to devise work plans and self-monitor, which correlates with higher performance. Conversely, a predominant MF is associated with more cautious, potentially passive or compliance-driven behaviors, focusing on avoiding errors or criticism, which can lead to a lack of initiative or enthusiasm in preventive work [42], potentially manifesting as less positive attitudes toward IAD prevention. Consistent with our hypothesis and previous research [88], this study confirmed a positive correlation between nurses' overall achievement motivation and their attitudes toward IAD prevention: Higher achievement motivation was associated with more positive preventive attitudes. This underscores the importance of motivational factors in shaping nurses' engagement with crucial patient safety practices.

The expression and impact of these achievement motivation orientations on attitudes appear to be deeply intertwined with the satisfaction of BPNs for autonomy, competence, and relatedness, as outlined by SDT [140]. Specifically, nurses predominantly driven by MS tend to exhibit more proactive and positive IAD prevention attitudes [141]. This proactive stance is likely catalyzed when their work environment supports their BPNs: Satisfaction of autonomy allows them to own and personalize prevention strategies; competence fulfillment reinforces their capability to achieve successful outcomes; and strong relatedness provides a supportive context for embracing challenges [142]. In such needs-supportive conditions, MS is channeled into autonomous motivation, where IAD prevention is viewed as a valued opportunity for mastery and positive impact. Conversely, a dominant MF orientation may lead to more cautious or reactive attitudes, particularly when BPNs are frustrated. Thwarted autonomy, low perceived competence, or a nonsupportive (low relatedness) environment can exacerbate the fear of failure, fostering controlled motivation (e.g., compliance merely to avoid censure) or amotivation, thereby leading to less engaged or even negative attitudes toward IAD prevention [143]. This framework also illuminates how the perceived responsibility for outcomes is appraised: as a stimulating challenge by MS-dominant nurses in needs-supportive settings, versus a potential threat by MF-dominant nurses when needs are unmet [144, 145].

Beyond the immediate channeling of MS and MF, SDT provides a crucial mechanism—internalization—for understanding the development of more robust and stable IAD prevention attitudes [146]. When nurses consistently experience satisfaction of their autonomy, competence, and relatedness while engaging in IAD prevention tasks, they are more likely to internalize the value of these behaviors, irrespective of their initial primary achievement driver (MS or MF) [147]. This internalization process shifts the locus of motivation from extrinsic factors or simple achievement striving toward personal endorsement and integration of preventive values with their professional identity (i.e., identified or integrated regulation) [148]. For instance, an MF-driven nurse, initially meticulous due to fear of error, may come to genuinely value meticulous prevention if her efforts are met with autonomy support, competence affirmation, and team appreciation. Such internalized motivation leads to attitudes that are not only positive but also deeply held, resilient, and less dependent on immediate external feedback, forming a stable foundation for consistent, high-quality preventive action because it becomes “the right thing to do” as a healthcare professional [68]. Thus, the formation of nurses' IAD prevention attitudes can be understood as a dynamic interplay where achievement motivation provides an initial impetus, but the quality and sustainability of these attitudes are fundamentally shaped by the extent to which the work environment satisfies their BPN.

### 4.3. Factors Influencing Nurses' Attitudes Toward IAD Prevention

In this study, position, department, number of participants in training, satisfaction with the work atmosphere, and achievement motivation were found to be the factors influencing nurses' attitudes toward IAD prevention through univariate analysis and binary logistic regression analysis. Nurse managers demonstrated more proactive IAD prevention attitudes compared to other clinical nurses. This can be understood from an SDT perspective: Managerial roles often entail greater autonomy, affording them more agencies in decision-making and shaping practice. These roles also provide more opportunities for external learning and conference attendance, which directly enhance their professional competence [149, 150]. As departmental role models, managers shoulder significant responsibility; when this is coupled with recognition and support from the organization, it satisfies their need for relatedness and affirms their competence, fostering a stronger sense of self-regulation and internalized commitment to best practices like IAD prevention, reflecting more autonomous motivation [151].

ICU nurses also exhibited more proactive attitudes, consistent with prior research [88]. The critical nature of ICU patients necessitates a high level of vigilance and skill. Targeted IAD training, common in ICUs, directly builds competence in this area [152]. The heightened sense of responsibility for safety and the need for astute patient assessment in this high-stakes environment likely foster a strong sense of professional competence. Furthermore, within well-functioning ICU teams, the shared focus on patient safety goals can significantly enhance feelings of relatedness [153]. This contrasts with general wards where lower perceived IAD risk might lead to fewer opportunities to develop specialized competence or to readily observe the immediate positive impact of preventive actions, thus potentially resulting in less autonomously motivated attitudes [154]. We acknowledge, however, that the relatively small sample of emergency and ICU nurses in this study may limit the generalizability of this specific departmental comparison.

Participation in a greater number of wound/ostomy/incontinence care training sessions was strongly associated with more proactive IAD prevention attitudes. Training directly addresses the need for competence by imparting essential knowledge and practical skills regarding IAD prevention and management, which in turn reduces anxiety and bolsters confidence [155, 156]. Consistent with the Knowledge–Attitude–Practice (KAP) model [19, 157], acquiring knowledge builds competence. When training further explains the rationale behind practices, it supports autonomy by fostering understanding and personal endorsement. If delivered in a supportive and collaborative manner, training also enhances relatedness among colleagues. Such holistically designed training is more likely to lead to the internalization of positive attitudes and the development of autonomous motivation toward IAD prevention. Our finding that only a small percentage of nurses attended three or more training sessions suggests a significant opportunity to enhance competence across the broader nursing population.

A high level of satisfaction with the work atmosphere also correlated positively with proactive IAD prevention attitudes. A positive work atmosphere typically signifies an environment where nurses experience greater relatedness, feeling respected, supported, and meaningfully connected with colleagues and superiors. Such environments often concurrently provide greater autonomy support and offer ample opportunities for nurses to exercise and develop their competence [158]. When these BPNs are satisfied, nurses are more inclined to cherish their profession, exhibit stronger commitment, perceive fewer negative emotions, and willingly embrace responsibilities, all of which reflect higher quality, autonomous motivation. Conversely, dissatisfaction with the work atmosphere often signals frustrated BPNs, leading to feelings of discontent, diminished professional value, and a shift toward controlled motivation or even amotivation; this naturally translates to less positive attitudes toward demanding tasks like IAD prevention [159].

Finally, nurses with higher achievement motivation demonstrated more positive attitudes toward IAD prevention. As discussed in Section 4.2, achievement motivation, particularly the MS, aligns with autonomous motivation when BPNs are supported. The satisfaction derived from tangible success in IAD prevention serves as a powerful affirmation of competence. The ability to direct one's efforts effectively in these tasks is an expression of autonomy. Furthermore, positive team collaboration in prevention efforts enhances relatedness. These experiences can continuously fuel the achievement drive, leading to a willingness to dedicate effort and embrace challenges in IAD prevention. Conversely, a dominant motivation to avoid failure, especially in an environment that thwarts BPNs, can erode enthusiasm and lead to more passive or reactive attitudes. These findings reinforce the understanding that individual motivational orientations interact significantly with the work context's ability to satisfy BPNs in shaping professional attitudes and engagement.

## 5. Implications for Nursing Management

The findings underscore the imperative for nursing management to cultivate work environments that systematically satisfy nurses' BPNs for autonomy, competence, and relatedness, as outlined by SDT. This approach should be nuanced, with interventions tailored to the distinct characteristics of different nurse profiles identified in this study. For nurses in profiles exhibiting lower overall attitudes or less experience (often corresponding to the “Lower Overall Attitude” profile), strategies should prioritize building competence and fostering relatedness. This can be achieved through clear, structured training on IAD prevention practices, evidence-based guidelines [160], and effective communication skills [161], potentially incorporating self-leadership techniques like goal-setting to systematically enhance professional skills [162, 163]. Concurrently, establishing robust mentorship programs with experienced, supportive mentors can provide crucial clinical guidance, constructive feedback, and positive role modeling, thereby enhancing both skill acquisition and a sense of belonging [164, 165]. Assisting these nurses in setting specific, achievable goals for IAD prevention, coupled with regular, informative feedback on their progress, will further build self-efficacy, paving the way for the gradual introduction of greater autonomy as their confidence and capabilities grow [166–169]. The aim of such interventions is to transition their motivation from being primarily externally regulated toward a more internalized form by fostering the necessary confidence and perceived value in preventive practices, which is critical for long-term engagement and mitigating early career burnout.

For experienced or higher-ranking nurses who may exhibit high general knowledge but lower personal responsibility (akin to the “Higher General Attitude with Lower Personal Responsibility” profile), interventions should focus on enhancing autonomy, clarifying and empowering personal accountability, and leveraging their existing competence for meaningful contributions. This involves clearly defining their leadership roles and specific responsibilities in IAD prevention, emphasizing individual accountability and the expectation to champion best practices [170]. Granting these nurses greater autonomy in designing, implementing, and leading IAD prevention quality improvement projects, supported by necessary resources and authority, can foster a profound sense of ownership and responsibility [171–173]. If these nurses are in supervisory roles, providing them with training in autonomy-supportive leadership styles can enable them to motivate their teams effectively, which in turn can reinforce their own sense of responsibility and leadership efficacy [172, 174, 175]. Furthermore, helping them connect their roles and individual contributions to broader organizational goals and positive patient outcomes can strengthen their sense of purpose and relatedness to a larger mission [176–178]. It is crucial to establish supportive, rather than punitive, accountability mechanisms that focus on learning and continuous improvement [179, 180]. For this group, lower personal responsibility may stem from a perceived disconnect between their capabilities and opportunities to make a significant impact; thus, empowerment and highlighting the tangible influence of their actions are key to re-igniting their sense of personal accountability, shifting their focus from hierarchical accountability to a professional responsibility for quality care and team performance. Ultimately, all developmental initiatives, including training, should be imbued with core SDT principles—providing clear rationales, acknowledging nurses' perspectives, offering choices where feasible, supporting competence through active learning, and fostering relatedness—as the *process* of these interventions is as critical as the content for facilitating the internalization of values and ensuring sustained improvements in IAD prevention. This requires a systemic commitment from leadership to create and maintain a truly needs-supportive organizational culture.

## 6. Limitations

This study has the following limitations. First, the study employed a cross-sectional design, collecting all data at a single point in time. While this allowed for the identification of associations between variables, it precludes the establishment of causal relationships. The directionality of the observed associations cannot be definitively determined from this design, and reciprocal relationships may exist. Future longitudinal studies are recommended to explore these dynamics over time and to provide stronger evidence for causal inferences. Secondly, the study's exclusive reliance on self-report data presented a potential risk for CMB. This concern was proactively addressed through both procedural control strategies implemented during data collection and rigorous statistical tests conducted in the analysis phase. These comprehensive assessments indicated that CMB did not exert a significant influence on our study's findings, although its potential presence cannot be entirely discounted with self-report methodology. Third, the sample size of this study was limited to nurses from a tertiary hospital in Fujian Province, which may restrict the generalizability of the results. Fourth, due to the relatively small salary differences among nurses in the tertiary hospital, salary was not included as a potential influencing factor, which may lead to discrepancies with existing research findings. Fifth, current research suggests that factors such as perceived social support, patient safety competence, and occupational identity may be related to nurses' attitudes toward the prevention of clinical adverse events. However, this study only examined the relationship between nurses' attitudes toward IAD prevention and achievement motivation. Therefore, future studies should consider a multicenter, large-sample approach to explore the relationship between various factors and nurses' attitudes toward IAD prevention.

## 7. Conclusions

This study employed a LPA to assess the preventive attitudes of nurses toward IAD. The results revealed a distinct categorization of nurses' preventive attitudes, which could be classified into two latent profiles: a low-level group and a high-level-low-personal-responsibility group. A multiple-factor analysis demonstrated that position, department, number of participants in the training, satisfaction with the work atmosphere, and achievement motivation are significant factors influencing nurses' preventive attitudes toward IAD. Furthermore, the study found a correlation between nurses' preventive attitudes toward IAD and their achievement motivation, wherein nurses with a positive attitude tend to strive for success, whereas those with a relatively negative attitude tend to avoid failure, reflecting two fundamentally different approaches to coping. This research contributes to improving nurses' preventive attitudes toward IAD, a pressing issue at present. Nursing managers should not only provide support from an environmental and policy perspective but also prioritize enhancing nurses' achievement motivation, promoting their subjective initiative, and fostering their self-directed learning consciousness.

## Figures and Tables

**Table 1:** The model fit indices for LPA.

Indicators	Criteria
AIC	The more accurately the model is classified, the smaller the value
BIC	The more accurately the model is classified, the smaller the value
aBIC	The more accurately the model is classified, the smaller the value
Entropy	Entropy over 0.8 indicates that 90% of individuals have been correctly classified
VLMR-LRT	When the *p* value is less than 0.05, it means that the class K model is better than the class K-1 model
BLRT	When the *p* value is less than 0.05, it means that the class K model is better than the class K-1 model
Latent class probability	Models were categorized more sensibly when they had a probability of at least 5% for each group

Abbreviations: aBIC, adjusted Bayesian Information Criteria; AIC, Akaike Information Criteria; BIC, Bayesian Information Criteria; BLRT, bootstrap likelihood ratio test; VLMR-LRT, Vuong–Lo–Mendell–Rubin likelihood ratio test.

**Table 2:** Demographic characteristics of nurses (*n* = 1058).

Variables	*n* (%)
Gender	
Female	1018 (96.2)
Male	40 (3.8)
Age (years)	
≤ 25	120 (11.3)
26∼35	523 (49.4)
36∼45	331 (31.3)
≥ 46	84 (7.9)
Professional title	
Nurse	114 (10.8)
Nurse practitioner	296 (28.0)
Nurse supervisor	559 (52.8)
Deputy chief nurse and above	89 (8.4)
Position	
Clinical nurse	970 (91.7)
Educational nurse	51 (4.8)
Head nurse	37 (3.5)
Specialized nurse	
Wound ostomy continence specialist nurse	8 (0.8)
Other specialized nurses	139 (13.1)
Staff nurse	911 (86.1)
Highest education	
Junior college and below	190 (18.0)
Undergraduate	849 (80.20)
Master's degree or above	19 (1.8)
Department	
Surgery	425 (40.2)
Internal medicine	492 (46.5)
Emergency department	45 (4.3)
ICU	96 (9.1)
Years of nursing work (years)	
≤ 2	103 (9.7)
3∼5	127 (12.0)
6∼10	356 (33.7)
11∼20	325 (30.7)
> 20	147 (13.9)
Are you a member of a wound/ostomy/incontinence team?	
Yes	66 (6.2)
No	992 (93.8)
Have you attended any wound/ostomy/incontinence care training?	
No	381 (36.0)
Yes	677 (64.0)
The number of times you have attended wound/ostomy/incontinence care training	
0	319 (30.2)
1∼2	614 (58.0)
3-5	76 (7.2)
≥ 6	49 (4.6)
The way you participate in wound/ostomy/incontinence care training	
Theoretical training	546 (51.6)
Practical training	14 (1.3)
Theory combined with practical training	207 (19.6)
No training	291 (27.5)
Number of study visits per year	
0	910 (86.0)
1∼2	142 (13.4)
> 2	6 (0.6)
Satisfaction with the working environment	
Dissatisfaction	21 (2.0)
General satisfaction	364 (34.4)
Satisfaction	673 (63.6)
Satisfaction with work atmosphere	
Dissatisfaction	9 (0.9)
General satisfaction	244 (23.1)
Satisfaction	805 (76.1)
Work satisfaction	
Dissatisfaction	13 (1.2)
General satisfaction	301 (28.4)
Satisfaction	744 (70.3)

**Table 3:** The latent profile analysis of nurses' preventive attitude toward IAD, fitting indicators of each model.

Model	AIC	BIC	aBIC	Entropy	VLMR-LRT	BLRT	Latent class probability
1	31023.038	31162.034	31073.101				
**2**	**28245.681**	**28459.139**	**28322.564**	**0.972**	**<** **0.001**	**<** **0.001**	**0.63422, 0.36578**
3	26859.993	27147.913	26963.695	0.963	0.068	< 0.001	0.03686, 0.60681, 0.35633
4	26372.579	26734.961	26503.101	0.936	< 0.001	< 0.001	0.03686, 0.60113, 0.35066, 0.01134

*Note:* The bolding highlights Model 2 as a key model in the latent profile analysis (LPA) due to its favorable performance across multiple evaluation dimensions: Model Fit Indices (AIC, BIC, and aBIC): The AIC (28245.681), BIC (28459.139), and aBIC (28322.564) for Model 2 are substantially lower than those of Model 1, indicating better data fit. Compared with more complex models (Model 3 and Model 4), these indices also strike a favorable balance between fit and model complexity. Classification Clarity (Entropy): Model 2's Entropy (0.972) is close to 1, which reflects high clarity in class membership—meaning the distinction between latent classes is well defined. Model comparison tests (VLMR–LRT and BLRT): Both the Vuong–Lo–Mendell–Rubin Likelihood Ratio Test –(VLMR–LRT, p < 0.001) and Bootstrap Likelihood Ratio Test (BLRT, p < 0.001) show that Model 2 (a 2-class model) significantly outperforms the 1-class model in fitting the data. Thus, bolding emphasizes Model 2's prominence for interpreting the latent profile patterns of nurses' preventive attitude toward IAD.

**Table 4:** Average probability of attribution for each latent profile.

Class	Profile 1	Profile 2
Profile 1	0.987	0.013
Profile 2	0.026	0.974

**Table 5:** Nurses' attitudes toward IAD prevention scores on each dimension (*n* = 1058).

Dimension	Number of items	Score ($\overset{¯}{X}$ ± *s*)		*t*	*p*
		Profile 1	Profile 2		
Impact	5	9.22 ± 1.97	13.18 ± 1.78	−33.413	< 0.001
Team responsibility	3	5.22 ± 1.17	7.25 ± 1.05	−27.562	< 0.001
Personal responsibility	3	2.85 ± 1.34	1.73 ± 1.72	10.992	< 0.001
Effectiveness of prevention	3	4.66 ± 1.30	5.63 ± 1.66	−9.8110	< 0.001
Total score	14	22.02 ± 4.12	27.81 ± 3.61	−22.974	< 0.001

**Table 6:** Nurses' attitudes toward IAD prevention score for each item (*n* = 1058).

Item	Score ($\overset{¯}{X}$ ± *s*)		Dimension
	Profile 1	Profile 2	
1. Why should I make an effort to prevent IAD if my colleagues don't?	1.83 ± 0.71	2.61 ± 0.75	Impact
2. IAD is just a simple skin problem and those lesions heal quickly	1.92 ± 0.54	2.69 ± 0.53	Impact
3. IAD, if it's nothing more than that …	1.72 ± 0.57	2.45 ± 0.75	Impact
4. It doesn't really matter if a patient develops IAD. IAD heals rather quickly	2.02 ± 0.51	2.93 ± 0.25	Impact
5. I quite understand that my colleagues show less interest in preventing IAD	1.71 ± 0.55	2.49 ± 0.72	Impact
6. In general, my team doesn't really consider IAD as an important issue	1.86 ± 0.50	2.89 ± 0.34	Team responsibility
7. My colleagues don't seem to pay much attention to IAD	1.81 ± 0.58	2.82 ± 0.40	Team responsibility
8. I invest more time in preventing IAD compared to my colleagues	1.59 ± 0.58	1.53 ± 0.79	Team responsibility
9. I will inform the responsible medical doctor if a patient suffers from IAD	0.94 ± 0.54	0.58 ± 0.70	Personal responsibility
10. If many patients suffer from IAD, I will discuss this with the senior nurse	0.80 ± 0.50	0.42 ± 0.67	Personal responsibility
11. I will discuss each IAD case with my colleagues	1.10 ± 0.59	0.73 ± 0.72	Personal responsibility
12. You can't prevent skin breakdown if a patient is incontinent	1.70 ± 0.63	2.26 ± 0.88	Effectiveness of prevention
13. If a patient is at risk to develop IAD, we won't be able to prevent it (even if we apply the best products we have)	1.85 ± 0.55	2.44 ± 0.75	Effectiveness of prevention
14. IAD incidence is an indicator of the quality of care	1.10 ± 0.57	0.92 ± 0.84	Effectiveness of prevention

**Table 7:** Nurses' achievement motivation scores on each dimension (*n* = 1058).

Variables	Correlation value	IAD prevention attitude score	Achievement motivation scores
Pearson's correlation coefficient			
IAD prevention attitude score	*R*	1	0.96
	*p*		0.002
Achievement motivation scores	*r*	0.96	1
	*p*	0.002	

**Table 8:** Correlation analysis of IAD prevention attitude scores and achievement motivation scores.

Dimension	Number of items	Score ($\overset{¯}{X}$ ± *s*)		*t*	*p*
		Profile 1	Profile 2		
Motivation for success	15	38.72 ± 6.74	41.88 ± 8.01	−6.837	< 0.001
Motivation to avoid failure	15	39.42 ± 7.36	38.24 ± 8.49	2.280	0.023
Total score	30	−0.69 ± 9.68	3.64 ± 11.99	−6.063	< 0.001

**Table 9:** Univariate analysis of latent profile.

Project	Variable	Profile 1	Profile 2	*Χ* ^2^/*t*	*p*
Gender	Female	647	371	0.21	0.647
	Male	24	16		

Age (years)	≤ 25	67	53	3.711	0.294
	26∼35	340	183		
	36∼45	209	122		
	≥ 46	55	29		

Professional title	Nurse	60	54	6.661	0.046
	Nurse practitioner	187	109		
	Nurse supervisor	366	193		
	Deputy chief nurse and above	31	58		

Position	Clinical nurse	620	350	9.582	0.008
	Educational nurse	36	15		
	Head nurse	15	22		

Specialized nurse	Wound ostomy continence specialist nurse	3	5	2.346	0.309
	Other specialized nurses	89	50		
	Staff nurse	579	332		

Highest education	Junior college and below	118	72	0.359	0.836
	Undergraduate	540	309		
	Master's degree or above	13	6		

Department	Surgery	291	134	17.656	0.001
	Internal medicine	309	183		
	Emergency department	26	19		
	ICU	45	51		

Years of nursing work (years)	≤ 2	52	51	9.139	0.028
	3∼5	78	49		
	6∼10	263	93		
	11∼20	181	144		
	> 20	97	50		

Are you a member of a wound/ostomy/incontinence team?	Yes	35	31	3.276	0.070
	No	636	356		

Have you attended any wound/ostomy/incontinence care training?	No	261	120	6.630	0.010
	Yes	410	267		

The number of times you have attended wound/ostomy/incontinence care training	0	223	96	22.824	< 0.001
	1∼2	384	230		
	3∼5	58	18		
	≥ 6	6	43		

The way you participate in wound/ostomy/incontinence care training	Theoretical training	330	216	14.077	0.003
	Practical training	6	8		
	Theory combined with practical training	126	81		
	No training	209	82		

Number of study visits per year	0	582	328	1.078	0.583
	1∼2	86	56		
	> 2	3	3		

Satisfaction with the working environment	Dissatisfaction	15	6	25.217	< 0.001
	General satisfaction	267	97		
	Satisfaction	389	284		

Satisfaction with work atmosphere	Dissatisfaction	7	2	40.544	< 0.001
	General satisfaction	196	48		
	Satisfaction	468	337		

Work satisfaction	Dissatisfaction	9	4	41.503	< 0.001
	General satisfaction	236	65		
	Satisfaction	426	318		

Achievement motivation scores		−0.69 ± 9.68	3.64 ± 11.99	−6.063	< 0.001

**Table 10:** Independent variable assignment table.

Independent variable	
Professional title	1 = nurse, 2 = nurse practitioner, 3 = nurse supervisor, 4 = deputy chief nurse and above
Position	1 = clinical nurse, 2 = educational nurse, 3 = head nurse
Department	1 = surgery, 2 = internal medicine, 3 = emergency department, 4 = ICU
Years of nursing work (years)	1 = ≤ 2, 2 = 3∼5, 3 = 6∼10, 4 = 11∼20, 5 = > 20
Have you attended any wound/ostomy/incontinence care training?	0 = no, 1 = yes
The number of times you have attended wound/ostomy/incontinence care training	1 = 0, 2 = 1∼2, 3 = 3∼5, 4 = ≥ 6
The way you participate in wound/ostomy/incontinence care training	1 = theoretical training, 2 = practical training, 3 = theory combined with practical training, 4 = no training
Satisfaction with the working environment	1 = dissatisfaction, 2 = general satisfaction, 3 = satisfaction
Satisfaction with work atmosphere	1 = dissatisfaction, 2 = general satisfaction, 3 = satisfaction
Work satisfaction	1 = dissatisfaction, 2 = general satisfaction, 3 = satisfaction

**Table 11:** Multifactorial analysis of nurses' attitudes toward IAD prevention by binary logistic regression.

Influencing factors	*β*	SE	Wald *X*^2^	*p*	OR	95% CI
Professional title (nurse as the control)	−0.147	0.163	0.813	0.367	0.863	0.628∼1.188
Position (clinical nurse as the control)	0.338	0.166	4.136	0.042	1.402	1.012∼1.941
Department (surgery as the control)	0.250	0.076	10.713	0.001	1.284	1.106∼1.492
Years of nursing work (years)	−0.050	0.110	0.204	0.651	0.951	0.786∼1.181
Have you attended any wound/ostomy/incontinence care training?	−0.162	0.193	0.700	0.403	0.851	0.583∼1.242
The number of times you have attended wound/ostomy/incontinence care training	0.285	0.193	4.999	0.025	1.329	1.036∼1.706
The way you participate in wound/ostomy/incontinence care training (theoretical training as the control)	−0.85	0.066	1.690	0.194	0.918	0.807∼1.044
Satisfaction with the working environment	0.055	0.186	0.088	0.767	1.057	0.733∼1.523
Satisfaction with work atmosphere	0.635	0.264	5.792	0.016	1.887	1.125∼3.165
Work satisfaction	0.288	0.260	0.775	0.379	1.257	0.756∼2.090
Achievement motivation scores	0.034	0.007	25.898	< 0.001	1.035	1.021∼1.048
Constant	−3.510	0.684	26.314	< 0.001	0.030	

## Data Availability

The study's original contributions are available in the article and supporting information. For additional inquiries, please contact the corresponding author(s).

## Supporting Information

STROBE Statement—checklist of items that should be included in reports of observational studies.

## References

[1] Sofoklis K., Dan L. B., David V. (2020). Knowledge Gaps in the Etiology and Pathophysiology of Incontinence-Associated Dermatitis: A Scoping Review. *The Journal of Wound, Ostomy and Continence Nursing: Official Publication of The Wound, Ostomy and Continence Nurses Society.*.

[2] Mikel G., Dimitri B., Donna Z. B. (2012). Incontinence-Associated Dermatitis: A Comprehensive Review and Update. *The Journal of Wound, Ostomy and Continence Nursing: Official Publication of The Wound, Ostomy and Continence Nurses Society.*.

[3] Gray M., Bliss D. Z., Doughty D. B., Ermer-Seltun J., Kennedy-Evans K. L., Palmer M. H. (2007). Incontinence-Associated Dermatitis: A Consensus. *The Journal of Wound, Ostomy and Continence Nursing*.

[4] Coyer F., Campbell J. (2018). Incontinence-Associated Dermatitis in the Critically Ill Patient: An Intensive Care Perspective. *Nursing in Critical Care*.

[5] Bliss D. Z., Savik K., Harms S., Fan Q., Wyman J. F. (2006). Prevalence and Correlates of Perineal Dermatitis in Nursing Home Residents. *Nursing Research*.

[6] Junkin J., Selekof J. L. (2007). Prevalence of Incontinence and Associated Skin Injury in the Acute Care Inpatient. *The Journal of Wound, Ostomy and Continence Nursing*.

[7] Kayser S. A., Phipps L., VanGilder C. A., Lachenbruch C. (2019). Examining Prevalence and Risk Factors of Incontinence-Associated Dermatitis Using the International Pressure Ulcer Prevalence Survey. *The Journal of Wound, Ostomy and Continence Nursing*.

[8] Völzer B., El Genedy-Kalyoncu M., Fastner A. (2023). Prevalence and Associations of Xerosis Cutis, Incontinence-Associated Dermatitis, Skin Tears, Pressure Ulcers, and Intertrigo in Aged Nursing Home Residents: A Representative Prevalence Study. *International Journal of Nursing Studies*.

[9] Campbell J. L., Coyer F. M., Osborne S. R. (2014). Incontinence‐Associated Dermatitis: A Cross‐Sectional Prevalence Study in the Australian Acute Care Hospital Setting. *International Wound Journal*.

[10] Zhang Q., Zhang K., Wang Y. (2021). Psychometric Properties of the Chinese Version of the Attitude Towards the Prevention of Incontinence-Associated Dermatitis (C-APrIAD) Among Chinese Nurses. *Journal of Tissue Viability*.

[11] Tay C., Yuh A. S., Sheau Lan E. L., Ong C. E., Aloweni F., Lopez V. (2020). Development and Validation of the Incontinence Associated Dermatitis Knowledge, Attitude and Practice Questionnaire. *Journal of Tissue Viability*.

[12] Lim Y. S. L., Carville K. (2019). Prevention and Management of Incontinence-Associated Dermatitis in the Pediatric Population: An Integrative Review. *The Journal of Wound, Ostomy and Continence Nursing*.

[13] Raepsaet C., Fourie A., Van Hecke A., Verhaeghe S., Beeckman D. (2020). Management of Incontinence‐Associated Dermatitis: A Systematic Review of Monetary Data. *International Wound Journal*.

[14] Beeckman D., Verhaeghe S., Defloor T., Schoonhoven L., Vanderwee K. (2011). A 3-in-1 Perineal Care Washcloth Impregnated With Dimethicone 3% Versus Water and pH Neutral Soap to Prevent and Treat Incontinence-Associated Dermatitis: A Randomized, Controlled Clinical Trial. *The Journal of Wound, Ostomy and Continence Nursing*.

[15] Zhang Y., Zhang P., Liu J. E., Gao F. (2021). A Qualitative Study on the Experience and Training Needs of ICU Nurses for Incontinence-Associated Dermatitis. *Advances in Skin & Wound Care*.

[16] Etafa W., Argaw Z., Gemechu E., Melese B. (2018). Nurses’ Attitude and Perceived Barriers to Pressure Ulcer Prevention. *BMC Nursing*.

[17] Grimshaw J., Eccles M., Tetroe J. (2004). Implementing Clinical Guidelines: Current Evidence and Future Implications. *Journal of Continuing Education in the Health Professions*.

[18] Van Damme N., Van Hecke A., Himpens A., Verhaeghe S., Beeckman D. (2019). Design and Psychometric Testing of the Attitude Towards the Prevention of Incontinence-Associated Dermatitis Instrument (APrIAD). *International Wound Journal*.

[19] Giacchero Vedana K. G., Magrini D. F., Zanetti A. C. G., Miasso A. I., Borges T. L., dos Santos M. A. (2017). Attitudes Towards Suicidal Behaviour and Associated Factors Among Nursing Professionals: A Quantitative Study. *Journal of Psychiatric and Mental Health Nursing*.

[20] Ajzen I., Fishbein M. The Influence of Attitudes on Behavior.

[21] Doll J., Mentz M., Orth B. (1991). Prediction of Goal-Directed Behavior: Attitude, Subjective Behavioral Competence and Emotions. *Zeitschrift fur Experimentelle und Angewandte Psychologie*.

[22] Moore Z., Price P. (2004). Nurses’ Attitudes, Behaviours and Perceived Barriers Towards Pressure Ulcer Prevention. *Journal of Clinical Nursing*.

[23] Rekisso A. D., Mengistu Z., Wurjine T. H. (2022). Nurses’ Attitudes Towards the Nursing Profession and Associated Factors in Selected Public Hospitals, Addis Ababa, Ethiopia, 2021: A Cross-Sectional Study. *BMC Nursing*.

[24] Afewerk S., Tesfaye E., Kebede A., Endeshaw D. (2023). Attitude and Its Associated Factors Towards the Nursing Profession Among BSc Nursing Students Learning at Governmental Universities in Amhara Region, Northwest Ethiopia, 2021. *International Journal of Africa Nursing Sciences*.

[25] Nyirenda M., Mukwato P. (2017). Job Satisfaction and Attitudes Towards Nursing Care Among Nurses Working at Mzuzu Central Hospital in Mzuzu, Malawi. *Malawi Medical Journal*.

[26] Huryk L. A. (2010). Factors Influencing Nurses’ Attitudes Towards Healthcare Information Technology. *Journal of Nursing Management*.

[27] Blau A., Sela Y., Grinberg K. (2023). Public Perceptions and Attitudes on the Image of Nursing in the Wake of COVID-19. *International Journal of Environmental Research and Public Health*.

[28] Barreto M. D. S., Marquete V. F., Camparoto C. W., García‐Vivar C., Barbieri‐Figueiredo M. D C., Marcon S. S. (2022). Factors Associated With Nurses’ Positive Attitudes Towards Families’ Involvement in Nursing Care: A Scoping Review. *Journal of Clinical Nursing*.

[29] Gassas R., Salem O. (2022). Factors Affecting Nurses’ Professional Values: A Comprehensive Integrative Review. *Nurse Education Today*.

[30] Sönmez M., Gürlek Kısacık Ö., Korkmaz S. (2025). Turkish Nurses’ Knowledge, Attitudes and Practices in the Management of Incontinence Associated Dermatitis: A Descriptive and Cross-Sectional Study. *Journal of Tissue Viability*.

[31] Asiri S., Alzahrani N., Alharbi H. F., Ayoub M. (2024). Nurses’ Knowledge Regarding Incontinence-Associated Dermatitis. *Risk Management and Healthcare Policy*.

[32] Richter M., Mazeres F., Brinkmann K. (2021). Clarity of Task Difficulty Moderates the Impact of the Explicit Achievement Motive on Physical Effort in Hand Grip Tasks. *PLoS One*.

[33] Cadenas-Sanchez C., Lamoneda J., Huertas-Delgado F. J. (2021). Association of Cardiorespiratory Fitness With Achievement Motivation in Physical Education in Adolescents. *International Journal of Environmental Research and Public Health*.

[34] Mahdavi P., Valibeygi A., Moradi M., Sadeghi S. (2023). Relationship Between Achievement Motivation, Mental Health and Academic Success in University Students. *Community Health Equity Research & Policy*.

[35] Tuominen H., Juntunen H., Niemivirta M. (2020). Striving for Success But at What Cost? Subject-Specific Achievement Goal Orientation Profiles, Perceived Cost, and Academic Well-Being. *Frontiers in Psychology*.

[36] Emamjomeh S. M., Toghyani A., Bahrami M. (2021). The Relationship Between Nursing Students’ Quality of Life With Their Academic Achievement Motivation in Isfahan University of Medical Sciences, Iran. *Iranian Journal of Nursing and Midwifery Research*.

[37] Bakker A. B., van Wingerden J. (2021). Do Personal Resources and Strengths Use Increase Work Engagement? The Effects of a Training Intervention. *Journal of Occupational Health Psychology*.

[38] Brewer C. S., Kovner C. T., Greene W., Cheng Y. (2009). Predictors of RNs’ Intent to Work and Work Decisions 1 Year Later in a U.S. National Sample. *International Journal of Nursing Studies*.

[39] DeLoach R., Monroe J. (2004). Job Satisfaction Among Hospice Workers: What Managers Need to Know. *The Health Care Manager*.

[40] Hsu H., Chen S., Yu H., Lou J. (2010). Job Stress, Achievement Motivation and Occupational Burnout Among Male Nurses. *Journal of Advanced Nursing*.

[41] Engin E., Cam O. (2009). Validity and Reliability Study of the Turkish Psychiatric Nurses of Job Motivation Scale. *Journal of Psychiatric and Mental Health Nursing*.

[42] Toode K., Routasalo P., Suominen T. (2011). Work Motivation of Nurses: A Literature Review. *International Journal of Nursing Studies*.

[43] Suominen T., Leino-Kilpi H., Merja M., Doran D., Puukka P. (2001). Staff Empowerment in Finnish Intensive Care Units. *Intensive and Critical Care Nursing*.

[44] Yıldız Z., Ayhan S., Erdoğmuş Ş. (2009). The Impact of Nurses’ Motivation to Work, Job Satisfaction, and Sociodemographic Characteristics on Intention to Quit Their Current Job: An Empirical Study in Turkey. *Applied Nursing Research*.

[45] Huang L., Liu M., Wang X., Hsu M. (2024). Interventions to Support the Psychological Empowerment of Nurses: A Scoping Review. *Frontiers in Public Health*.

[46] Jiang J., Liu M., Li Y., Gao H., Tian L. (2024). Nursing Professionalism: A Scoping Review of Implementation Level, Evaluation Instruments, Influential Factors, and Intervention Strategies. *Journal of Nursing Management*.

[47] Dai X., Xie C., Wu Y., Chen T., Lu F. (2025). Factors Associated With Burnout Among Chinese Operating Room Nurses: A Meta-Analysis. *BMC Nursing*.

[48] Pang D., Senaratana W., Kunaviktikul W., Klunklin A., McElmurry B. J. (2009). Nursing Values in China: The Expectations of Registered Nurses. *Nursing and Health Sciences*.

[49] Su L., Wichaikhum O., Abhicharttibutra K. (2022). Predictors of Organizational Commitment Among Chinese Nurses During the COVID‐19 Pandemic. *International Nursing Review*.

[50] Tzeng H.-M. (2002). The Influence of Nurses’ Working Motivation and Job Satisfaction on Intention to Quit: An Empirical Investigation in Taiwan. *International Journal of Nursing Studies*.

[51] Wang X., Liu M., Leung A. Y. M. (2025). Nurses’ Self-Efficacy, Job Embeddedness, and Psychological Empowerment: A Cross-Sectional Study. *Journal of Nursing Management*.

[52] Dan X., Huang Y., Ren J.-H., Tian Y.-L., He Y.-L. (2023). Professional Nursing Practice Environment and Work Engagement: The Mediating Roles of Self-Efficacy and Achievement Motivation. *Journal of Nursing Research*.

[53] Qiu L., Miao Q., Zhao Y., Zhu L., Wang Y. (2023). A Study on the Impact of Life Meaning on Occupational Identity of Nursing Students in China: A Cross-Sectional Study. *BMC Nursing*.

[54] Sriratanaprapat J., Songwathana P. (2011). Nurses’ Job Satisfaction Within the Context of Asian Cultures: A Concept Analysis. *Pacific Rim International Journal of Nursing Research*.

[55] Lee J. J., Yang S. C. (2019). Professional Socialisation of Nursing Students in a Collectivist Culture: A Qualitative Study. *BMC Medical Education*.

[56] Liou S.-R., Tsai H.-M., Cheng C.-Y. (2013). Acculturation, Collectivist Orientation and Organisational Commitment Among Asian Nurses Working in the US Healthcare System. *Journal of Nursing Management*.

[57] Zou J., Zhu X., Fu X. (2025). The Experiences of Organizational Silence Among Nurses: A Qualitative Meta-Synthesis. *BMC Nursing*.

[58] Cook D. A., Artino A. R. (2016). Motivation to Learn: An Overview of Contemporary Theories. *Medical Education*.

[59] Ganotice F. A., Chan L., Chow A. Y. M. (2022). What Characterize High and Low Achieving Teams in Interprofessional Education: A Self-Determination Theory Perspective. *Nurse Education Today*.

[60] Deci E. L., Ryan R. M. (1985). The General Causality Orientations Scale: Self-Determination in Personality. *Journal of Research in Personality*.

[61] Deci E. L., Ryan R. M. (2000). The “What” and “Why” of Goal Pursuits: Human Needs and the Self-Determination of Behavior. *Psychological Inquiry*.

[62] Gagné M., Deci E. L. Self‐Determination Theory and Work Motivation.

[63] Ntoumanis N. (2002). Motivational Clusters in a Sample of British Physical Education Classes. *Psychology of Sport and Exercise*.

[64] Koestner R., Losier G. F. (2002). Distinguishing Three Ways of Being Highly Motivated: A Closer Look at Introjection, Identification, and Intrinsic Motivation. *Handbook of Self-detErmination Research*.

[65] Wang C. K. J., Biddle S. J. H. (2001). Young People’s Motivational Profiles in Physical Activity: A Cluster Analysis. *Journal of Sport & Exercise Psychology*.

[66] Howard J., Gagné M., Morin A. J. S., Van den Broeck A. (2016). Motivation Profiles at Work: A Self-Determination Theory Approach. *Journal of Vocational Behavior*.

[67] Sheldon K. M., Elliot A. J. (1998). Not all Personal Goals are Personal: Comparing Autonomous and Controlled Reasons for Goals as Predictors of Effort and Attainment. *Personality and Social Psychology Bulletin*.

[68] Ryan R. M., Deci E. L. (2000). Self-Determination Theory and the Facilitation of Intrinsic Motivation, Social Development, and Well-Being. *American Psychologist*.

[69] Ryan R. M., Deci E. L. (2017). *Self-Determination Theory: Basic Psychological Needs in Motivation, Development, and Wellness*.

[70] Ross B. M., Barnes D. M. (2018). Self-Determination Theory With Application to Employee Health Settings. *Workplace Health & Safety*.

[71] Baard P. P., Deci E. L., Ryan R. M. (2004). Intrinsic Need Satisfaction: A Motivational Basis of Performance and Weil-Being in Two Work Settings. *Journal of Applied Social Psychology*.

[72] Bono J. E., Judge T. A. (2003). Self-Concordance at Work: Toward Understanding the Motivational Effects of Transformational Leaders. *Academy of Management Journal*.

[73] Greguras G. J., Diefendorff J. M. (2010). Why Does Proactive Personality Predict Employee Life Satisfaction and Work Behaviors? A Field Investigation of the Mediating Role of the Self-Concordance Model. *Personnel Psychology*.

[74] Moran C. M., Diefendorff J. M., Kim T.-Y., Liu Z.-Q. (2012). A Profile Approach to Self-Determination Theory Motivations at Work. *Journal of Vocational Behavior*.

[75] Deci E. L., Flaste R. (1996). *Why We Do What We Do: Understanding Self-Motivation*.

[76] Sheldon K. M., Williams G., Joiner T. (2008). *Self-Determination Theory in the Clinic: Motivating Physical and Mental Health*.

[77] Riley B. H., McDermott R. C. (2018). Applying Self-Determination Theory to Adolescent Sexual-Risk Behavior and Knowledge: A Structural Equation Model. *Journal of the American Psychiatric Nurses Association*.

[78] Johnson V. D. (2007). Promoting Behavior Change: Making Healthy Choices in Wellness and Healing Choices in Illness—Use of Self-Determination Theory in Nursing Practice. *Nursing Clinics of North America*.

[79] Teutsch D., Frick E., Kubitza J. (2025). What Motivates Critical Care Nurses to Stay in Their Job?–Structural Aspects for Empowering Intrinsic Motivation in Permissive Professional Contexts: A Scoping Review. *Intensive and Critical Care Nursing*.

[80] Ogrim G., Kropotov J., Brunner J. F., Candrian G., Sandvik L., Hestad K. A. (2014). Predicting the Clinical Outcome of Stimulant Medication in Pediatric Attention-Deficit/Hyperactivity Disorder: Data From Quantitative Electroencephalography, Event-Related Potentials, and a Go/No-Go Test. *Neuropsychiatric Disease and Treatment*.

[81] Taylor G., Jungert T., Mageau G. A. (2014). A Self-Determination Theory Approach to Predicting School Achievement Over Time: The Unique Role of Intrinsic Motivation. *Contemporary Educational Psychology*.

[82] Duprez V., van der Kaap-Deeder J., Beeckman D., Verhaeghe S., Vansteenkiste M., Van Hecke A. (2020). Nurses’ Interaction Styles When Supporting Patients in Self-Management: A Profile Approach. *International Journal of Nursing Studies*.

[83] Hao Y., Bai C., Wang T. (2024). Gratitude and Depressive Symptoms in Chinese Nurses: From a Self-Determination Theory Perspective. *Worldviews on Evidence-Based Nursing*.

[84] Onyishi I. E., Enwereuzor I. K., Ogbonna M. N., Ugwu F. O., Amazue L. O. (2019). Role of Career Satisfaction in Basic Psychological Needs Satisfaction and Career Commitment of Nurses in Nigeria: A Self-Determination Theory Perspective. *Journal of Nursing Scholarship*.

[85] Hosseini L. J., Rafiemanesh H., Bahrami S. (2022). Levels of Motivation and Basic Psychological Need Satisfaction in Nursing Students: In Perspective of Self-Determination Theory. *Nurse Education Today*.

[86] Zhang K. M., Tulloch H. E., Brunet J. (2020). What Motivates Nurses to Exercise? Determinants of Physical Activity Among Canadian Nurses Using Self-Determination Theory. *Annals of Behavioral Medicine*.

[87] Bergman L. R., Magnusson D. (1997). A Person-Oriented Approach in Research on Developmental Psychopathology. *Development and Psychopathology*.

[88] Mengying Q., Yexiang F., Ping L., Zhen L. (2023). A Study on the Attitude and Influencing Factors of Preventing Incontinence-Associate Dermatitis Among ICU Nurse. *The Journal of Nursing Administration*.

[89] Wang L., Wang H., Yang D., Dai F. (2019). Investigation on Influencing Factors for Knowledge Attitude Practice Regarding Incontinence Associated Dermatitis in ICU Nurses. *Chinese General Practice Nursing*.

[90] Li H., Wang D., Chen Z. (2020). Investigation of Knowledge, Attitudes and Practice of Incontinence-Associated Dermatitis in ICU Nurses in Hainan Province. *Hainan Medicine*.

[91] Oberski D. (2016). Mixture Models: Latent Profile and Latent Class Analysis. *Modern Statistical Methods for HCI*.

[92] Kong L., Zhang H. (2023). Latent Profile Analysis of Depression in Non-Hospitalized Elderly Patients With Hypertension and Its Influencing Factors. *Journal of Affective Disorders*.

[93] Lanza S. T., Tan X., Bray B. C. (2013). Latent Class Analysis With Distal Outcomes: A Flexible Model-Based Approach. *Structural Equation Modeling: A Multidisciplinary Journal*.

[94] Li Z., Lynch J., Sun T., Rizkyana Q., Cheng J. T., Benson A. J. (2024). Power Motives, Personality Correlates, and Leadership Outcomes: A Person-Centered Approach. *Journal of Personality*.

[95] Chen C., Zheng X., Cui Y. (2024). Heterogeneity of Work Alienation and Its Relationship With Job Embeddedness Among Chinese Nurses: A Cross-Sectional Study Using Latent Profile Analysis. *BMC Nursing*.

[96] Zheng X., Liu H., Zhang M. (2024). Understanding the Mechanism of Safety Attitude Mitigates the Turnover Intention Novice Nurses Via the Person-Centred Method: A Theory-Driven, Deductive Cross-Sectional Study. *Journal of Advanced Nursing*.

[97] von Elm E., Altman D. G., Egger M., Pocock S. J., Gøtzsche P. C., Vandenbroucke J. P. (2008). The Strengthening the Reporting of Observational Studies in Epidemiology (STROBE) Statement: Guidelines for Reporting Observational Studies. *Journal of Clinical Epidemiology*.

[98] Weber E. (1977). Kendall, M.: Multivariate Analysis. Charles Griffin B Co. LTD. London, High Wycombe 1975. 210 S., 9 Abb., 27 Tab., 1 Anhang, £ 6,80. *Biometrical Journal*.

[99] Howard M. C., Hoffman M. E. (2018). Variable-Centered, Person-Centered, and Person-Specific Approaches: Where Theory Meets the Method. *Organizational Research Methods*.

[100] Nylund K. L., Asparouhov T., Muthén B. O. (2007). Deciding on the Number of Classes in Latent Class Analysis and Growth Mixture Modeling: A Monte Carlo Simulation Study. *Structural Equation Modeling*.

[101] Jin J., Wang J., Xing L., Fan L. (2020). Attitude Towards the Prevention of Incontinence-Associated Dermatitis Scale in ICU Nurses: Translation, and Reliability and Validity Testing. *Nursing Journal of Chinese People’s Liberation Army*.

[102] Yu M. (2023). *The Reliability and Validity of the Translated Chinese Version of the Attitude Towards the Prevention of Incontinence-Associated Dermatitis Instrument*.

[103] Nygård R., Gjesme T. (1973). Assessment of Achievement Motives: Comments and Suggestions. *Scandinavian Journal of Educational Research*.

[104] Ye R., Hagtvet K. A. (1992). Measurement and Analysis of Achievement Motivation. *Psychological Development and Education*.

[105] Cao H., Wang J., Wang L., Ding A. (2017). Study on the Correlation Between Emotional Intelligence and Achievement Motivation of Nurses During the Training Period. *Journal of Nurses Training*.

[106] Ding Q. Study on the Correlation Between Achievement Motivation, Resourcefulness, and Job Burnout in Emergency Nurses.

[107] YuWang Y.Y., Gao X., Ma Q., Cheng L., Jing Z., Tingting L. (2020). Analysis of the Correlation Between Career Anchor and Achievement Motivation of Newly Recruited Operating Room Nurses. *Hospital Administration Journal of Chinese People’s Liberation Army*.

[108] Leung C. L. K., Li K.-K., Wei V. W. I. (2022). Profiling Vaccine Believers and Skeptics in Nurses: A Latent Profile Analysis. *International Journal of Nursing Studies*.

[109] Lanza S. T., Rhoades B. L., Nix R. L., Greenberg M. T. (2010). Modeling the Interplay of Multilevel Risk Factors for Future Academic and Behavior Problems: A Person-Centered Approach. *Development and Psychopathology*.

[110] Lubke G., Muthén B. O. (2007). Performance of Factor Mixture Models as a Function of Model Size, Covariate Effects, and Class-Specific Parameters. *Structural Equation Modeling*.

[111] Podsakoff P. M., MacKenzie S. B., Lee J. Y., Podsakoff N. P. (2003). Common Method Biases in Behavioral Research: A Critical Review of the Literature and Recommended Remedies. *Journal of Applied Psychology*.

[112] Han Y., Kim J. S., Seo Y. (2020). Cross-Sectional Study on Patient Safety Culture, Patient Safety Competency, and Adverse Events. *Western Journal of Nursing Research*.

[113] Tai C., Chen D., Zhang Y., Teng Y., Li X., Ma C. (2024). Exploring the Influencing Factors of Patient Safety Competency of Clinical Nurses: A Cross-Sectional Study Based on Latent Profile Analysis. *BMC Nursing*.

[114] Al-Mugheed K., Bayraktar N., Al-Bsheish M. (2022). Patient Safety Attitudes Among Doctors and Nurses: Associations With Workload, Adverse Events, Experience. *Healthcare (Basel, Switzerland)*.

[115] Beele H., Smet S., Van Damme N., Beeckman D. (2018). Incontinence-Associated Dermatitis: Pathogenesis, Contributing Factors, Prevention and Management Options. *Drugs & Aging*.

[116] Dissemond J., Assenheimer B., Gerber V. (2021). Moisture-Associated Skin Damage (MASD): A Best Practice Recommendation From Wund-D.A.CH. *JDDG: Journal der Deutschen Dermatologischen Gesellschaft*.

[117] Van Damme N., Clays E., Verhaeghe S., Van Hecke A., Beeckman D. (2018). Independent Risk Factors for the Development of Incontinence-Associated Dermatitis (Category 2) in Critically Ill Patients With Fecal Incontinence: A Cross-Sectional Observational Study in 48 ICU Units. *International Journal of Nursing Studies*.

[118] Zhou Y., Wu Y., Gu X. (2017). A Study on the Application of Feedforward Control in Prevention of Incontinence-Associated Dermatitis in Patients With Acute Severe Poisoning. *The Journal of Nursing Administration*.

[119] Yuanyuan X., Shi G., Zhang Y. (2021). Evidence-Based Practice in Prevention of Fecal Incontinence Associated Dermatitis in ICU Patients. *Chinese Journal of Nursing*.

[120] Griffin M. A., Neal A., Parker S. K. (2007). A New Model of Work Role Performance: Positive Behavior in Uncertain and Interdependent Contexts. *Academy of Management Journal*.

[121] Hu Q., Balwi M. K. B. M. (2025). The Development of Ethical Codes for Social Work in China: Opportunities and Challenges. *Ethics and Social Welfare*.

[122] Corrigan P. W., Lee E.-J. (2021). Family-Centered Decision Making for East Asian Adults With Mental Illness. *PS*.

[123] Villamin P., Lopez V., Thapa D. K., Cleary M. (2025). Retaining a Multicultural Nursing Workforce: A Self-Determination Theory Perspective. *Journal of Transcultural Nursing*.

[124] Liu Y., Choi W.-S., Wang W., Kang S.-W. (2025). The Impact of Self-Sacrificial Leadership on Employee Creativity: A Moderated Mediation Model in the Post-Pandemic Chinese Service Sector. *Behavioral Sciences*.

[125] Ye J., Mao A., Wang J. (2020). From Twisting to Settling Down as a Nurse in China: A Qualitative Study of the Commitment to Nursing as a Career. *BMC Nursing*.

[126] Zhao J., Wang B.-L., Qin X., Liu Y., Liu T. (2024). Core Elements of Excellent Hospital Leadership: Lessons From the Five Top-Performing Hospitals in China. *International Journal for Quality in Health Care*.

[127] Oshodi T. O., Bruneau B., Crockett R., Kinchington F., Nayar S., West E. (2019). Registered Nurses’ Perceptions and Experiences of Autonomy: A Descriptive Phenomenological Study. *BMC Nursing*.

[128] Zhu H., Xu C., Jiang H., Li M. (2021). A Qualitative Study on the Experiences and Attributions for Resigned Nurses With Career Plateau. *International Journal of Nursing Science*.

[129] Hunter K., Cook C. (2018). Role-Modelling and the Hidden Curriculum: New Graduate Nurses’ Professional Socialisation. *Journal of Clinical Nursing*.

[130] Samuriwo R., Dowding D. (2014). Nurses’ Pressure Ulcer Related Judgements and Decisions in Clinical Practice: A Systematic Review. *International Journal of Nursing Studies*.

[131] Brewer M. B., Chen Y. R. (2007). Where (Who) are Collectives in Collectivism? Toward Conceptual Clarification of Individualism and Collectivism. *Psychological Review*.

[132] Oyserman D., Lee S. W. S. (2008). Does Culture Influence What and How We Think? Effects of Priming Individualism and Collectivism. *Psychological Bulletin*.

[133] Boysen P. G. (2013). Just Culture: A Foundation for Balanced Accountability and Patient Safety. *The Ochsner Journal*.

[134] Krys K., Vignoles V. L., de Almeida I., Uchida Y. (2022). Outside the “Cultural Binary”: Understanding Why Latin American Collectivist Societies Foster Independent Selves. *Perspectives on Psychological Science*.

[135] Kitayama S., Salvador C. E. (2024). Cultural Psychology: Beyond East and West. *Annual Review of Psychology*.

[136] Balante J., Broek D. V. D., White K. (2021). How Does Culture Influence Work Experience in a Foreign Country? An Umbrella Review of the Cultural Challenges Faced by Internationally Educated Nurses. *International Journal of Nursing Studies*.

[137] Fish J. A., Sharplin G., Wang L., An Y., Fan X., Eckert M. (2022). Cross-Cultural Differences in Nurse Burnout and the Relationship With Patient Safety: An East-West Comparative Study. *Journal of Advanced Nursing*.

[138] Chen L., Lan H., Fu X., Peng L. (2015). Study on the Influence of Psychological Capital and Achievement Motivation on ICU Male Nurses’ Vocational Delay of Gratification. *Chinese Nursing Management*.

[139] Guan Y., Yuan J. (2013). Influences of Samyaksmrti on Chievement Motivation in Nurses of Rheumatism Immunity Departmen. *The Journal of Nursing Administration*.

[140] Tanaka A., Yamauchi H. (2001). A Model for Achievement Motives, Goal Orientations, Intrinsic Interest, and Academic Achievement. *Psychological Reports*.

[141] Sayed R. I. E., Ali M. R. (2017). Achievement Motivation and Its Relation to Nurses’ Decision Making Believes, Ability, and Job Burnout at Obstetric and Gynecological Departments. *Clinical Nursing Studies*.

[142] St-Cyr J., Vallerand R. J., Chénard-Poirier L. A. (2021). The Role of Passion and Achievement Goals in Optimal Functioning in Sports. *International Journal of Environmental Research and Public Health*.

[143] Elliot A. J., Sheldon K. M. (1997). Avoidance Achievement Motivation: A Personal Goals Analysis. *Journal of Personality and Social Psychology*.

[144] Grinberg E., Nachoum R., Kanat-Maymon Y. (2025). The Effect of Basic Psychological Needs on Healthcare Professionals’ Work-Related Psychological Outcomes. *Work*.

[145] Veenstra G. L., Dabekaussen K. F. A. A., Molleman E., Heineman E., Welker G. A. (2022). Health Care Professionals’ Motivation, Their Behaviors, and the Quality of Hospital Care: A Mixed-Methods Systematic Review. *Health Care Management Review*.

[146] Patrick H., Williams G. C. (2012). Self-Determination Theory: Its Application to Health Behavior and Complementarity With Motivational Interviewing. *International Journal of Behavioral Nutrition and Physical Activity*.

[147] Deci E. L., Eghrari H., Patrick B. C., Leone D. R. (1994). Facilitating Internalization: The Self-Determination Theory Perspective. *Journal of Personality*.

[148] Ryan R. M. (1995). Psychological Needs and the Facilitation of Integrative Processes. *Journal of Personality*.

[149] Tangeraas Hansen M. J., Storm M., Syre H., Dalen I., Husebø A. M. L. (2023). Attitudes and Self-Efficacy Towards Infection Prevention and Control and Antibiotic Stewardship Among Nurses: A Mixed-Methods Study. *Journal of Clinical Nursing*.

[150] Ünver S., Fındık Ü. Y., Özkan Z. K., Sürücü Ç. (2017). Attitudes of Surgical Nurses Towards Pressure Ulcer Prevention. *Journal of Tissue Viability*.

[151] Suvarnakich P., Montrikul Na Ayudhaya B. (2022). Compliance With the Ethical Competence Framework by Head Nurses. *Nursing Ethics*.

[152] Zhang Y., Liu J., Chang X. (2021). Development and Application of Management Information System for the Recognition and Prevention of Incontinence-Associated Dermatitis. *Chinese Journal of Nursing*.

[153] Wu C., He C., Yan J. (2024). Patterns of Information Literacy and Their Predictors Among Emergency Department Nurses: A Latent Profile Analysis Based on the Person-Context Interaction Theory. *BMC Nursing*.

[154] Qiao H., Li B., Li W., Niu A., Huang Y. (2020). Comparison of Anxiety, Depression, and Job Burnout of Nurses in Intensive Care Unit to Nurses in General Ward. *Chinese Mental Health Journal*.

[155] Travers C., Henderson A., Graham F., Beattie E. (2018). Turning Education Into Action: Impact of a Collective Social Education Approach to Improve Nurses’ Ability to Recognize and Accurately Assess Delirium in Hospitalized Older Patients. *Nurse Education Today*.

[156] del Cotillo-Fuente M., Valls-Matarín J., Sandalinas-Mulero I. (2021). Efficacy of a Comprehensive Strategy to Reduce Moisture-Associated Skin Damage in an Intensive Care Unit: A Quasi-Experimental Study. *Intensive and Critical Care Nursing*.

[157] Wan T. T. H., Rav-Marathe K., Marathe S. (2016). A Systematic Review of Kap-O Framework for Diabetes. *Medical Research Archives*.

[158] Mao A., Wang J., Zhang Y., Cheong P. L., Van I. K., Tam H. L. (2020). Factors Influencing Recruitment and Retention of Male Nurses in Macau and Mainland China: A Collaborative, Qualitative Study. *BMC Nursing*.

[159] Chen D. (2019). Mediating Effect of Nurses’ Perceived Career Benefit Between Career Success and Occupational Commitment. *Journal of Nursing*.

[160] Omidi M., Meftahi H., Omidi N. (2018). The Relationship Between Organizational Citizen Behavior and Nursing Achievement Motivation. *Health Management & Information Science*.

[161] Li L. Z., Yang P., Singer S. J., Pfeffer J., Mathur M. B., Shanafelt T. (2024). Nurse Burnout and Patient Safety, Satisfaction, and Quality of Care: A Systematic Review and Meta-Analysis. *JAMA Network Open*.

[162] Oldland E., Redley B., Botti M., M Hutchinson A. (2023). Nurses’ Motivations and Desired Learning Outcomes of Postgraduate Critical Care Studies: A Descriptive Exploratory Study. *Australian Critical Care*.

[163] Pursio K., Kvist T., Kankkunen P., Fennimore L. A. (2025). Self-Leadership and Why It Matters to Nurses: A Scoping Review. *International Nursing Review*.

[164] Gong Z., Li M. (2022). Relationship Between Nursing Mentorship and Transformational Leadership of Mentor: A Cross-Sectional Study. *Journal of Nursing Management*.

[165] Mínguez Moreno I., González de la Cuesta D., Barrado Narvión M. J., Arnaldos Esteban M., González Cantalejo M. (2023). Nurse Mentoring: A Scoping Review. *Healthcare*.

[166] Mackintosh-Franklin D. C. (2021). An Evaluation of Formative Feedback and Its Impact on Undergraduate Student Nurse Academic Achievement. *Nurse Education in Practice*.

[167] Zhang Z.-J., Zhang C.-L., Zhang X.-G. (2015). Relationship Between Self-Efficacy Beliefs and Achievement Motivation in Student Nurses. *Chinese Nursing Research*.

[168] Finn C. P. (2001). Autonomy: An Important Component for Nurses’ Job Satisfaction. *International Journal of Nursing Studies*.

[169] Mrayyan M. T. (2004). Nurses’ Autonomy: Influence of Nurse Managers’ Actions. *Journal of Advanced Nursing*.

[170] Frögéli E., Rudman A., Gustavsson P. (2019). The Relationship Between Task Mastery, Role Clarity, Social Acceptance, and Stress: An Intensive Longitudinal Study With a Sample of Newly Registered Nurses. *International Journal of Nursing Studies*.

[171] Orgambídez A., Almeida H. (2020). Social Support, Role Clarity and Job Satisfaction: A Successful Combination for Nurses. *International Nursing Review*.

[172] Guibert‐Lacasa C., Vázquez‐Calatayud M. (2022). Nurses’ Clinical Leadership in the Hospital Setting: A Systematic Review. *Journal of Nursing Management*.

[173] Yesilbas H., Kantek F. (2024). Relationship Between Structural Empowerment and Job Satisfaction Among Nurses: A Meta-Analysis. *International Nursing Review*.

[174] James A. H., Bennett C. L., Blanchard D., Stanley D. (2021). Nursing and Values-Based Leadership: A Literature Review. *Journal of Nursing Management*.

[175] Wardani E., Ryan T., Yusuf M. (2021). How Nurses in a Developing Country Perceive and Experience Leadership: A Qualitative Study. *Contemporary Nurse*.

[176] Hagerty B. M., Patusky K. L. (2003). Reconceptualizing the Nurse-Patient Relationship. *Journal of Nursing Scholarship*.

[177] Strandås M., Bondas T. (2018). The Nurse–Patient Relationship as a Story of Health Enhancement in Community Care: A Meta-Ethnography. *Journal of Advanced Nursing*.

[178] Wei H. (2022). The Development of an Evidence-Informed Convergent Care Theory: Working Together to Achieve Optimal Health Outcomes. *International Journal of Nursing Science*.

[179] Leonenko M., Drach-Zahavy A. (2016). You are Either Out on the Court, or Sitting on the Bench: Understanding Accountability From the Perspectives of Nurses and Nursing Managers. *Journal of Advanced Nursing*.

[180] Chesterton L., Tetley J., Cox N., Jack K. (2021). A Hermeneutical Study of Professional Accountability in Nursing. *Journal of Clinical Nursing*.

