# Supplementary material for: Understanding the Relationship Between Chinese Nurses' Attitudes Toward Incontinence-Related Dermatitis and Achievement Motivation Through a Person-Centered Method: A Theory-Driven Cross-Sectional Study
Source: J Nurs Manag. 2025 Oct 29;2025:3381812. doi: 10.1155/jonm/3381812 (PMC12588752; doi:10.1155/jonm/3381812)
Supplement: Supporting Information — Additional supporting information can be found online in the Supporting Information section. [file 3381812.f1.docx]

STROBE Statement—checklist of items that should be included in reports of observational studies

|  | Item No. | Recommendation | Page  No. | Relevant text from manuscript |
| --- | --- | --- | --- | --- |
| **Title and abstract** | 1 | (*a*) Indicate the study’s design with a commonly used term in the title or the abstract | 1 | Data was collected using a general information questionnaire, the Attitude Towards the Prevention of Incontinence-Associated Dermatitis Instrument, and the Achievement Motivation Scale. Latent Profile Analysis (LPA) identified latent subgroups of attitudes, while t-tests, chi-square tests, and binary logistic regression analysis examined factors influencing these attitudes. |
|  |  | (*b*) Provide in the abstract an informative and balanced summary of what was done and what was found | 1 | Two subgroups emerged: the low-level group (63.42%) and the high-level, low-personal-responsibility group (36.57%). A significant correlation was found between nurses' attitudes and achievement motivation. Positive attitudes were associated with higher motivation for success, while negative attitudes were linked to motivation to avoid failure. Factors influencing attitudes included position, department, number of participants in wound/ostomy/incontinence care training, satisfaction with the work atmosphere, and achievement motivation scores. |
| Introduction | | | |  |
| Background/rationale | 2 | Explain the scientific background and rationale for the investigation being reported | 1-7 | Previous research on nurses' attitudes toward IAD prevention has primarily focused on investigating the current status of these attitudes. However, the existence of heterogeneity within nurses' attitudes toward IAD prevention remains an area that warrants further exploration. Both domestically and internationally, no specific studies have examined the relationship between nurses' attitudes toward IAD prevention and achievement motivation, and the deeper, more intricate mechanisms linking these two factors remain unclear. Additionally, most existing studies rely on standardized scale scores or threshold values to determine the level of nurses' attitudes toward IAD prevention. However, this "variable-centered" approach overlooks the variations introduced by individual differences. This method assumes that subgroups assessed using similar prevention attitudes are homogeneous, yet there is considerable heterogeneity in the responses within each subgroup, with individuals exhibiting different reaction patterns to each item despite having similar attitudes. Consequently, to some extent, the findings of these studies do not truly represent the diversity within the nursing population. Latent Profile Analysis (LPA), a person-centered approach, offers several advantages in this context. Firstly, it enables the identification of heterogeneity within the study population by classifying potential subgroups[47,48]. Nurses within each preventive attitude subgroup are homogeneous, while those across different subgroups exhibit heterogeneity. This approach provides a better understanding of the distinct characteristics of nurse populations with varying preventive attitudes. Secondly, LPA reduces the complexity and higher-order interactions of variables present in variable-centered methods, such as the need for larger sample sizes to detect effects and the uncertainty regarding how variables combine within individuals[49,50]. Thirdly, LPA has been successfully applied in numerous psychological trait studies, and tailored interventions can be developed for specific subgroups to better meet their unique needs[51,52]. Therefore, by applying LPA, this study aims to identify previously unobserved subgroups, thus enhancing our understanding of their hidden characteristics. These findings will contribute to the literature on nurses' attitudes toward IAD prevention and underscore the value of person-centered assessments for developing customized interventions. The results of this study may serve as a foundation for the creation of targeted intervention strategies. |
| Objectives | 3 | State specific objectives, including any prespecified hypotheses | 5-7 | This study proposes the following hypotheses:  **H1: There are subgroup differences in nurses' attitudes toward IAD prevention.**  **H2: Nurses' attitudes toward IAD prevention are associated with their achievement motivation.**  **H3: Nurses with a motivation for success exhibit more positive attitudes toward IAD prevention, whereas those with a motivation to avoid failure demonstrate less proactive attitudes in IAD prevention.** |
| Methods | | | |  |
| Study design | 4 | Present key elements of study design early in the paper | 7 | A cross-sectional study was carried out in 2024 between September and October. The STROBE (strengthening the reporting of observational research in epidemiology) statement was adhered to in this study (see Appendix S1). |
| Setting | 5 | Describe the setting, locations, and relevant dates, including periods of recruitment, exposure, follow-up, and data collection | 7 | A cross-sectional study was carried out in 2024 between September and October.Nurses were conveniently sampled from a tertiary comprehensive hospital in Xiamen City, Fujian Province, China. |
| Participants | 6 | (*a*) *Cohort study*—Give the eligibility criteria, and the sources and methods of selection of participants. Describe methods of follow-up  *Case-control study*—Give the eligibility criteria, and the sources and methods of case ascertainment and control selection. Give the rationale for the choice of cases and controls  *Cross-sectional study*—Give the eligibility criteria, and the sources and methods of selection of participants | 7 | Nurses were conveniently sampled from a tertiary comprehensive hospital in Xiamen City, Fujian Province, China. Inclusion criteria were: (a) completion of nurse registration and the National Nurse Licensure Examination (NNLE); (b) independent performance of nursing duties; and (c) willingness to participate in the study. Head nurses, nursing students, and nurses absent due to illness or leave during the study period were excluded. |
|  |  | (*b*) *Cohort study*—For matched studies, give matching criteria and number of exposed and unexposed  *Case-control study*—For matched studies, give matching criteria and the number of controls per case | - |  |
| Variables | 7 | Clearly define all outcomes, exposures, predictors, potential confounders, and effect modifiers. Give diagnostic criteria, if applicable | 7-9 | **Demographic questionnaire**  The general demographic characteristics data were collected using a questionnaire specifically designed by the researchers. The data included gender, age, professional title, position, specialized nurse, highest education, department, and years of nursing work, are you a member of a wound/ostomy/incontinence team? Have you attended any wound/ostomy/incontinence care training? The number of times you have attended wound/ostomy/incontinence care training, the way you participate in wound/ostomy/incontinence care training, the number of study visits per year, satisfaction with the working environment, satisfaction with the work atmosphere, and work satisfaction |
| Data sources/ measurement | 8* | For each variable of interest, give sources of data and details of methods of assessment (measurement). Describe comparability of assessment methods if there is more than one group | *7-9* | **Attitude Towards the prevention Incontinence-Associated Dermatitis instrument (APrIAD)**  The APrIAD is employed to assess nurses' attitudes towards the prevention of Incontinence-Associated Dermatitis (IAD), a tool developed by Van Damme[22] and adapted into Chinese by Jiayao Jin[57]. This instrument comprises four dimensions: (a) perceptions regarding the impact of IAD on patients (5 items), (b) beliefs about the team's responsibility in preventing IAD (3 items), (c) convictions about personal responsibility in preventing IAD (3 items), and (d) opinions on the efficacy of IAD prevention products and procedures (3 items). A 4-point Likert scale is utilized, with scores ranging from 0 to 3, representing 'strong disagreement' to 'strong agreement', and a total score of 0 to 42, where higher scores indicate a more positive attitude towards IAD prevention. The scale exhibiting good data fit (χ2 = 2810.769, χ2/ df = 2.197, CFI = 0.941, GFI = 0.936, CFI = 0.941, NFI = 0.921, RMSEA = 0.037) was subjected to a confirmatory factor analysis in previous studies. Chinese populations have validated the Scale. In Yu's[58] and Qi's[59] research, Cronbach's α was 0.774 and 0.809, respectively; in this study, it was 0.766 for the nursing environment.  **Achievement Motivation Scale (AMS)**  The AMS scale is utilized to measure nurses' achievement motivation. This scale was developed by Nygard[60] in 1973 and revised into Chinese by Chinese scholars Ye Renmin and Hagtvet [61]. It comprises two dimensions: the motivation for success (with 15 items) and the motivation to avoid failure (with 15 items), totaling 30 items. The Likert 4-point scoring system is employed, where scores from 1 to 4 correspond to "Strongly Disagree" to "Strongly Agree." The total score for each dimension ranges from 15 to 60 points. Achievement motivation score is calculated as the difference between the motivation for success score and the motivation to avoid failure score, with higher scores indicating stronger achievement motivation. The Cronbach's α coefficient of the scale in this study is 0.903. |
| Bias | 9 | Describe any efforts to address potential sources of bias | 10 | **Common method deviation test**  There can be some common method variance (CMV) because this study used self-report data. The reverse item approach, an anonymous questionnaire, and randomization of item assignment across different constructs were among the strategies we employed in this study to prevent CMV.  In this study, the effects of an unmeasured latent methods factor (ULMC) were controlled to investigate the existence of common method bias (CMB). A bifactor model that included a common method factor was tested based on the original model. Confirmatory factor analysis results showed that adding the common method factor to the original model resulted in negligible modifications (<0.05) in several fit indices (ΔRMSEA = 0.140, ΔSRMR = 0.113, ΔCFI = 0.642, ΔTLI = 0.595). We discovered that there is no significant CMB since the model did not considerably refine the fitting effect by controlling the common method components. |
| Study size | 10 | Explain how the study size was arrived at | 7 | The sample size was calculated as ten times the number of scale items, following Kendall's sample size estimation standard. This study included 44 items: the Chinese version of the Attitude Towards the Incontinence-Associated Dermatitis Scale (14 items) and the Achievement Motivation Scale (30 items). Therefore, the minimum required sample size was N = 10 * (30 + 14) = 440. Considering a 10% to 20% non-response rate, the final required sample size was N = 440 ÷ (1 - 20%) = 550. Prior research suggests that larger sample sizes are sufficient for LPA. Ultimately, 1058 questionnaires were collected, yielding a 100% response rate. |

Continued on next page

| Quantitative variables | 11 | Explain how quantitative variables were handled in the analyses. If applicable, describe which groupings were chosen and why | - |  |
| --- | --- | --- | --- | --- |
| Statistical methods | 12 | (*a*) Describe all statistical methods, including those used to control for confounding | 9-10 | To identify statistically significant differences among the preventive attitude subgroups, univariate analyses, including the t-test and chi-square test, were first conducted. Variables that were significant in the univariate analyses (p < 0.05, two-sided) were included in the binary logistic regression model. The cutoff for inclusion in the final model was set at p < 0.05. |
|  |  | (*b*) Describe any methods used to examine subgroups and interactions | 9-10 | For data analysis, this study employed Mplus 8.3 and SPSS 26.0. Kolmogorov-Smirnov was used to test the normality of the measurement data, and the enumeration data were expressed as n (%). Standard deviation (SD) and mean were used to represent normal data, whereas median and interquartile range were used to represent skewed data. The scores of each dimension of nurses' prevention attitude toward IAD were the main variables in our latent profile analysis, which we performed after first applying the normality and common method bias tests to the data. LPA is a statistical technique designed to evaluate individual characteristics based on multiple continuous variables. It uses probabilistic methods to determine the subgroup to which an individual most likely belongs, identifying patterns to categorize individuals into distinct groups. The optimal classification model was selected based on the practical significance of the profile and the quality of the fit indices, as shown in Table 1. Once the ideal number of latent profiles was determined, nurses were assigned to latent profile groups based on their most probable latent class membership. |
|  |  | (*c*) Explain how missing data were addressed | - |  |
|  |  | (*d*) *Cohort study*—If applicable, explain how loss to follow-up was addressed  *Case-control study*—If applicable, explain how matching of cases and controls was addressed  *Cross-sectional study*—If applicable, describe analytical methods taking account of sampling strategy | - |  |
|  |  | (*e*) Describe any sensitivity analyses | - |  |
| Results | | | | |
| Participants | 13* | (a) Report numbers of individuals at each stage of study—eg numbers potentially eligible, examined for eligibility, confirmed eligible, included in the study, completing follow-up, and analysed | 10 | The study sample consisted of 1,058 nurses |
|  |  | (b) Give reasons for non-participation at each stage | - |  |
|  |  | (c) Consider use of a flow diagram | - |  |
| Descriptive data | 14* | (a) Give characteristics of study participants (eg demographic, clinical, social) and information on exposures and potential confounders | 10-11 | The study sample consisted of 1,058 nurses, with a predominantly female population (96.2%, n = 1,018) and a smaller proportion of males (3.8%, n = 40). The majority of nurses were between 26 and 35 years old (49.4%, n = 523). In terms of job roles, 52.8% (n = 559) held supervisory positions, while 91.7% were clinical nurses. Wound ostomy continence specialist nurses accounted for only 0.8% (n = 8) of the sample, with 13.1% (n = 139) representing other specialized nurses. The highest qualification for most nurses was a bachelor's degree (80.2%, n = 849). Departmentally, 46.5% (n = 492) of nurses worked in medical departments, while 40.2% (n = 425) were from surgical departments. The largest proportion of nurses had 11-20 years of work experience (38.5%, n = 407), followed by those with 6-10 years of experience (25.9%, n = 274). Further details on participant characteristics are provided in **Table 2**. |
|  |  | (b) Indicate number of participants with missing data for each variable of interest | - |  |
|  |  | (c) *Cohort study*—Summarise follow-up time (eg, average and total amount) | - |  |
| Outcome data | 15* | *Cohort study*—Report numbers of outcome events or summary measures over time | *-* |  |
|  |  | *Case-control study—*Report numbers in each exposure category, or summary measures of exposure | *-* |  |
|  |  | *Cross-sectional study—*Report numbers of outcome events or summary measures | *12* | The results of the univariate analysis revealed significant differences in several variables across the two latent profiles: professional title, position, department, years of nursing work, participation in wound/ostomy/incontinence care training, training frequency, training method, work environment satisfaction, work atmosphere satisfaction, job satisfaction, and achievement motivation (see **Table 9**). |
| Main results | 16 | (*a*) Give unadjusted estimates and, if applicable, confounder-adjusted estimates and their precision (eg, 95% confidence interval). Make clear which confounders were adjusted for and why they were included | - |  |
|  |  | (*b*) Report category boundaries when continuous variables were categorized | - |  |
|  |  | (*c*) If relevant, consider translating estimates of relative risk into absolute risk for a meaningful time period | - |  |

Continued on next page

| Other analyses | 17 | Report other analyses done—eg analyses of subgroups and interactions, and sensitivity analyses | 11 | Four latent profile models in all explored in this work, **Table 3** shows the fitting indexes of the various profiles. The values of AIC, BIC, and aBIC rapidly decreased as the number of latent profiles increased. When the profile was divided into three categories, the VLMR-LRT was not statistically significant, suggesting that the two-profile and four-profile models performed better than the three-profile model. Since each category should make up at least 5.0% of the entire sample size, the four-profile model's profile had a very small proportion, which made it impracticable to use. Contrary to this, Model 2's entropy value is the highest. In Model 2, the two categories had average attribution probabilities of 98.7% and 97.4%, respectively (refer to Table 4). The category model's VLMR-LRT and BLRT were statistically significant, suggesting that Model 2 was the best fit. Given the extensive analysis described above, the best classification result for clinical nurses' attitudes toward IAD prevention was obtained by using a two-class solution.  **Naming categories of latent profiles of nurses' attitudes toward IAD prevention**  A chart was created based on the scores of the two nurse categories' attitudes toward IAD prevention on the 14 items, and Model 2 was chosen as the best model to identify the categories based on the scores of the two nurse categories on the 14 items (see **Figure 1**). Profile 1 accounted for 63.42% of the total, and because of its low item scores, it became known as the low-level group. Profile 2, with a share of 36.57%, had the highest scores for most items in this category. Among them, item 8 (“I invest more time in preventing IAD compared to my colleagues”), item 9 ("I will inform the responsible medical doctor if a patient suffers from IAD”), item 10 ("If many patients suffer from IAD, I will discuss this with the senior nurse"), item 11 ("I will discuss each IAD case with my colleagues"), and item 14 (“IAD incidence is an indicator of the quality of care”) scored the lowest. Among these items, items 9, 10, and 11 belong to the dimension of personal responsibility, reflecting the fact that such nurses believe that it is difficult for personal behavior to make a difference in the prevention of IAD. Therefore, it was named as high level-low personal responsibility group. |
| --- | --- | --- | --- | --- |
| Discussion | | | | |
| Key results | 18 | Summarise key results with reference to study objectives | 12-18 | The nurses' attitudes toward IAD prevention exhibit considerable heterogeneity, a crucial finding that emerges from the adoption of novel methodologies. Previous studies have characterized nurses' preventive attitudes at an aggregate level; while the findings and implications of these studies are by no means negligible, their limitations overlook the potential for stratification. This study employed a person-centered approach to identify heterogeneous attitudes toward IAD prevention among Chinese nurses. It further examined the association between demographic factors and identified attitude profiles and explored the relationship between these latent categories of IAD prevention attitudes and achievement motivation. To our knowledge, this is the first study to investigate nurses' attitudes towards IAD prevention using this methodological approach. |
| Limitations | 19 | Discuss limitations of the study, taking into account sources of potential bias or imprecision. Discuss both direction and magnitude of any potential bias | 19 | Considering the positive influence of achievement motivation on nurses' attitudes toward IAD prevention, the findings of this study provide valuable insights for nursing managers to implement measures that enhance nurses' prevention and care capabilities. Targeted training programs are crucial for improving nurses' knowledge, skills, and confidence in IAD care, while cultivating a supportive work environment that offers ample resources, reduces hierarchical barriers, and promotes peer learning through mentoring and knowledge sharing. Acknowledging proactive efforts and providing constructive feedback can further elevate nurses' self-efficacy and intrinsic motivation. Moreover, nursing managers should guide nurses with a high avoidance of failure motivation to recognize the relationship between opportunity and threat perception, transforming their tendency to avoid failure and emphasize threats into a more positive perception of opportunities. Encouraging nurses to attribute their achievements to internal factors, applying positive psychology in their work, and fully leveraging their intrinsic motivation will help them develop a correct value orientation and encourage their pursuit of personal growth. |
| Interpretation | 20 | Give a cautious overall interpretation of results considering objectives, limitations, multiplicity of analyses, results from similar studies, and other relevant evidence | 12-18 | This study employed a latent profile analysis to assess the preventive attitudes of nurses toward IAD. The results revealed a distinct categorization of nurses' preventive attitudes, which could be classified into two latent profiles: a low-level group and a high-level-low personal responsibility group. A multiple-factor analysis demonstrated that position, department, number of participants in the training, satisfaction with the work atmosphere, and achievement motivation are significant factors influencing nurses' preventive attitudes toward IAD. Furthermore, the study found a correlation between nurses' preventive attitudes towards IAD and their achievement motivation, wherein nurses with a positive attitude tend to strive for success, whereas those with a relatively negative attitude tend to avoid failure, reflecting two fundamentally different approaches to coping. This research contributes to improving nurses' preventive attitudes towards IAD, a pressing issue at present. Nursing managers should not only provide support from an environmental and policy perspective but also prioritize enhancing nurses' achievement motivation, promoting their subjective initiative, and fostering their self-directed learning consciousness. |
| Generalisability | 21 | Discuss the generalisability (external validity) of the study results | 19 | Considering the positive influence of achievement motivation on nurses' attitudes toward IAD prevention, the findings of this study provide valuable insights for nursing managers to implement measures that enhance nurses' prevention and care capabilities. Targeted training programs are crucial for improving nurses' knowledge, skills, and confidence in IAD care, while cultivating a supportive work environment that offers ample resources, reduces hierarchical barriers, and promotes peer learning through mentoring and knowledge sharing. Acknowledging proactive efforts and providing constructive feedback can further elevate nurses' self-efficacy and intrinsic motivation. Moreover, nursing managers should guide nurses with a high avoidance of failure motivation to recognize the relationship between opportunity and threat perception, transforming their tendency to avoid failure and emphasize threats into a more positive perception of opportunities. Encouraging nurses to attribute their achievements to internal factors, applying positive psychology in their work, and fully leveraging their intrinsic motivation will help them develop a correct value orientation and encourage their pursuit of personal growth. |
| Other information | |  | | |
| Funding | 22 | Give the source of funding and the role of the funders for the present study and, if applicable, for the original study on which the present article is based | Attached files | This study was supported by the Fujian Province health technology plan project (2024TG024); Xiamen Municipal Science and Technology Project (No. 3502Z20244ZD1078). |

*Give information separately for cases and controls in case-control studies and, if applicable, for exposed and unexposed groups in cohort and cross-sectional studies.

**Note:** An Explanation and Elaboration article discusses each checklist item and gives methodological background and published examples of transparent reporting. The STROBE checklist is best used in conjunction with this article (freely available on the Web sites of PLoS Medicine at http://www.plosmedicine.org/, Annals of Internal Medicine at http://www.annals.org/, and Epidemiology at http://www.epidem.com/). Information on the STROBE Initiative is available at www.strobe-statement.org.
